# Association of human-specific expanded short tandem repeats with neuron-specific regulatory features

**DOI:** 10.1126/sciadv.adp9707

**Published:** 2025-05-30

**Authors:** Qiming Liu, Weidong Tian

**Affiliations:** ^1^ State Key Laboratory of Genetics and Development of Complex Phenotypes, Department of Computational Biology, School of Life Sciences, Fudan University, Shanghai, China.; ^2^ Children’s Hospital of Fudan University, Shanghai, China.; ^3^ Children’s Hospital of Shandong University, Jinan, China.

## Abstract

Short tandem repeats (STRs), characterized by high–copy number mutations, represent one of the fastest-evolving genomic elements. However, human-specific expanded STRs (heSTRs) have lacked comprehensive genome-wide characterization. Leveraging 148 human and 26 nonhuman primate haploid genomes, we identified 8813 heSTRs with robust expansions in copy number distributions. Our analysis revealed notable associations between heSTRs and brain- and neuron-specific distal regulatory signals. Potential target genes regulated by heSTRs, identified by incorporating distal regulations, are enriched with neuronal development–related functions and disorders, displaying neuron-specific expression enhancement in humans. Moreover, heSTRs are associated with enhanced chromatin accessibility specifically in human neurons. In addition, heSTRs show substantial association with pathogenic STR loci exhibiting abnormal copy number variations, as reported by cohort studies on schizophrenia and autism. This study underscores the role of heSTRs in both human evolution and disorders, offering valuable insights for future research on STRs from an evolutionary perspective.

## Teaser

Human-specific expanded STRs shape neuronal traits and disorders through distal regulatory mechanisms.

## INTRODUCTION

Throughout evolution, humans and nonhuman primates (NHPs) have developed distinct traits (*1*), particularly in neuroanatomical features governing cognitive abilities (*2*, *3*). Given the minimal amino acid sequence differences between humans and NHPs (*4*, *5*), research has increasingly focused on the role of regulatory mutations in driving these phenotypic divergences (*6*). Early investigations into highly conserved regulatory elements in noncoding regions led to the identification of human accelerated regions (HARs) (*7*–*11*), which are strongly associated with neuronal functions. More recently, Mangan *et al.* (*12*) identified human ancestor quickly evolved regions (HAQERs) in noncoding regions that exhibit rapid divergence from NHPs and show associations with neuronal development. These findings underscore the role of accelerated evolution in noncoding regions in shaping human-specific phenotypes.

Short tandem repeats (STRs) are repetitive DNA sequences with unit lengths ranging from 1 to 6 base pairs (bp) primarily distributed in noncoding regions and constituting about 3% of the human genome (*13*). A defining characteristic of STRs is their high mutation rates, particularly in copy numbers, which are several orders of magnitude higher than single-nucleotide variations (*14*). This high mutation rate results in a high prevalence of copy number polymorphisms within human populations (*15*, *16*). Such polymorphisms have been linked to changes in gene expression (*17*, *18*), alternative splicing (*19*), and various neurological disorders (*20*, *21*), including Fragile X syndrome (*22*), Huntington’s disease (*23*), and amyotrophic lateral sclerosis (*24*). Beyond human populations, copy number variations of STRs are widespread across species (*25*) and have been implicated in interspecies divergence in gene expression (*26*), underscoring their potential evolutionary significance. However, the role of accelerated evolution in STRs and its impact on human phenotypic evolution remain largely unexplored.

Recent investigations by Kim *et al.* (*27*) and Sulovari *et al.* (*28*) have shed light on human-specific expanded tandem repeats, showcasing a notable correlation with altered expression patterns in the human brain or neurons. However, Kim *et al.* (*27*) exclusively focused on variable number tandem repeats (VNTRs) with repeat unit lengths exceeding 6 bp, thereby excluding STRs. In contrast, Sulovari *et al.* (*28*) focused on a subset of STRs identified using third-generation sequencing, specifically those newly resolved or exhibiting higher copy numbers than those in the hg38 reference genome. Despite these contributions, the study of Sulovari *et al.* (*28*) was limited by its small sample size, examining only six haploid genomes from humans and six from NHPs. This limited dataset may not provide a robust representation of STR copy number distributions. Furthermore, the NHP species were restricted to three great ape species closely related to humans, offering a narrow evolutionary perspective. This limitation raises the possibility that some identified STRs may not be strictly human specific. Given the extensive copy number variations of STRs within human populations and across primate species, a larger sample size and a more diverse evolutionary background are needed to enhance the reliability and robustness of identified STR expansion events.

In this study, we curated a comprehensive dataset of haploid genomes assembled using third-generation sequencing (*29*–*32*) to explore the accelerated evolution of STRs. This dataset includes 148 human genomes representing 27 geographically diverse human subpopulations and 26 genomes from seven NHP species, encompassing both closely and distantly related evolutionary lineages. By analyzing STRs with relatively conserved copy number distributions across the seven NHP species, we identified 8813 human-specific expanded STRs (heSTRs) exhibiting robust copy number expansion in humans.

Detailed analyses revealed that these heSTRs are enriched in brain-specific regulatory signals and associated with chromatin loops and innermost hierarchically topologically associating domains (ihTADs) specifically implicated in neuronal function. Genes potentially regulated by heSTRs show enrichment in functions related to neuronal development and exhibit enhanced expression in human neuronal cells. Furthermore, heSTRs were found to markedly overlap with pathogenic STR loci identified in schizophrenia and autism cohorts. Collectively, these findings underscore the potential role of heSTRs in shaping the evolution of human-specific phenotypes, offering valuable insights into the genetic mechanisms underlying human brain development and associated disorders.

## RESULTS

### Identification and characterization of heSTRs in the human genome

**Fig. 1.**
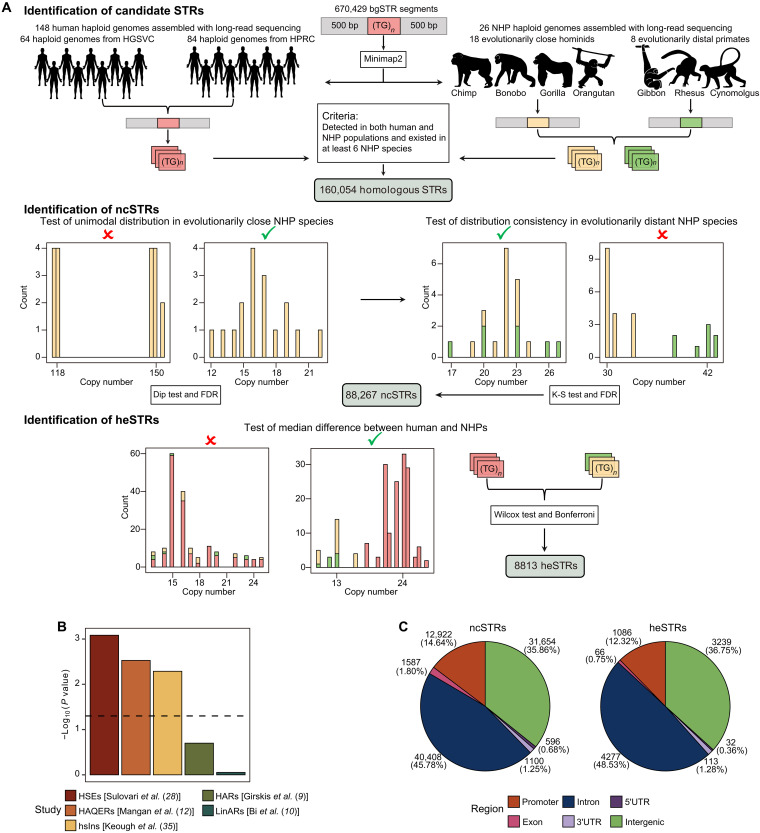
Genome-wide identification and characterization of heSTRs. (**A**) Computational pipeline illustrating the genome-wide identification process of heSTRs. See Materials and Methods for a detailed description. K-S test, Kolmogorov-Smirnov test. (**B**) Enrichment analysis displaying the overlaps of documented genomic regions indicative of accelerated evolution with heSTRs. *P* values were computed using the fisher.test function in R. (**C**) Distribution of genome regions for NHP-conserved STRs (ncSTRs) and heSTRs.

To identify heSTRs, we analyzed 174 haploid primate genomes assembled using third-generation sequencing. This dataset includes 148 human genomes, representing five superpopulations and 27 subpopulations. It also includes 26 NHP genomes, with 18 from four closely related great ape species and 8 from three more distantly related primate species. Detailed information about the curated haploid genomes is available in Materials and Methods and table S1. We began with a panel of 670,429 STRs annotated by RepeatMasker (*33*) on the hg38 reference genome, which served as our background STRs (bgSTRs). For each STR, we included 500-bp flanking regions and used Minimap2 (*34*) to map the sequence segment to each of the 174 haploid genomes. After identifying an STR segment in a haploid genome, we confirmed the presence of the same STR motif using RepeatMasker (*33*) and determined its copy number. We then selected STRs present in humans and at least six of the seven NHP species. This process resulted in 160,054 homologous STRs. The workflow for this analysis is shown in the top panel of Fig. 1A. Additional details about STR genotyping are provided in Materials and Methods.

We implemented a rigorous computational pipeline to identify heSTRs. First, we selected homologous STRs exhibiting copy number distributions approximating a unimodal pattern within the four great ape species closely related to humans. Next, we excluded STRs with copy number distributions in the three evolutionarily distant NHP species that deviated from this pattern. This step yields 88,267 STRs with consistent copy number distributions across all seven NHP species, referred to as NHP-conserved STRs (ncSTRs) (Fig. 1A, middle). We then conducted a one-tailed Wilcoxon test for each ncSTR to compare its copy numbers in 148 haploid human genomes against those in 26 haploid NHP genomes. This analysis led to the identification of 8813 heSTRs with significantly expanded copy numbers in humans (Bonferroni adjusted *P* < 0.05) (Fig. 1A, bottom, and table S2). In all subsequent comparisons between heSTRs and ncSTRs, we used the set of ncSTRs excluding heSTRs to ensure nonoverlapping groups.

In humans, heSTRs have a median length of 46 bp, with a median increase of 12 bp compared to those in NHPs (fig. S1, A and B). More than 78% of heSTRs exhibit a length increase of at least 20% compared to NHPs, while fewer than 10% show a greater than 100% increase in length (fig. S1, C and D). heSTRs are enriched for mono- and dinucleotide repeats, particularly (A)*_n_* [odds ratio (OR) = 1.29 and Fisher’s two-sided *P* = 3.62 × 10^−13^] and (AC)*_n_* (OR = 2.07 and *P* = 2.53 × 10^−233^) motifs (fig. S2, A and B). Compared to ncSTRs, heSTRs show significant enrichment at loci associated with accelerated evolution (refer to Fig. 1B for specific *P* values). These loci include human-specific expansions (HSEs) of tandem repeats (*28*), human-specific insertions (hsIns) (*35*), and HAQERs (*12*) (Fig. 1B). This enrichment at HAQERs and hsIns, which are known for their neuron-specific regulatory roles (*12*, *35*), implicates the potential regulatory functions of heSTRs. However, heSTRs were not linked to HARs (*9*) and human lineage-specific accelerated regions (LinARs) (*10*), which focus on regions under strong sequence conservation (Fig. 1B).

ncSTRs are predominantly located within noncoding regions, with a notable presence in promoter regions (Fig. 1C). In contrast, heSTRs exhibit a lower proportion in promoter regions but are more frequently found in intronic regions (Fig. 1C). Genome-wide distribution analysis revealed that, unlike human-specific VNTRs (motif length > 6 bp), which are enriched in subtelomeric regions (*28*), heSTRs are more evenly distributed across the genome and positioned farther from subtelomeric regions compared to ncSTRs (fig. S3, A and B). This discrepancy likely reflects the general depletion of STRs, as opposed to VNTRs, in subtelomeric regions (*15*, *36*). These differences between heSTRs and ncSTRs were statistically significant based on permutation testing (*P* < 0.05; fig. S4).

Because much of STR variation has been attributed to sequences derived from transposable elements (TEs) (*28*), we examined the relationship between heSTRs and TEs. Our analysis was constrained by our STR reference panel’s treatment of STRs and TEs as distinct elements, allowing us to focus only on the association between TEs and STR flanking regions. Analysis revealed that the 100-bp flanking regions of heSTRs harbored significantly (proportion test *P* = 4.26 × 10^−59^) more TEs compared to ncSTRs (fig. S5A), with notable enrichment of L1 (long interspersed nuclear element) and *Alu* (short interspersed nuclear element) elements (fig. S5B). These findings highlight both the inherent variability and expansion potential of TE-associated STRs while suggesting that retrotransposon transduction (*37*) may contribute to the emergence of some heSTRs.

### Association of heSTRs with neuron-specific regulatory features

To explore the potential regulatory role of heSTRs, we investigated their association with six types of candidate *cis*-regulatory elements (ccREs) identified by The Encyclopedia of DNA Elements (ENCODE) Project (*38*). These elements include representative deoxyribonuclease (DNase) I hypersensitive sites (rDHSs), which indicate general regulatory characteristics, promoter-like signatures (PLSs), proximal enhancer-like signatures (pELSs), distal enhancer-like signatures (dELS), and DNase-H3K4me3 and CTCF-only signatures, which represent poised elements or candidate insulators.

**Fig. 2.**
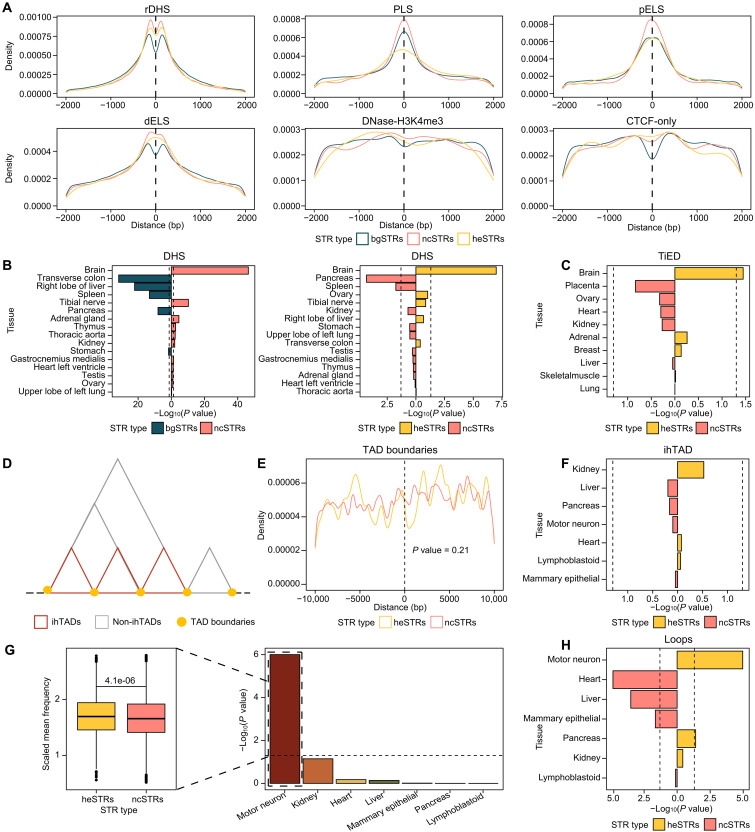
Profiling of heSTRs using various genomic features. (**A**) Association of STRs with six types of ccREs classified by the ENCODE project. The densities of STRs relative to the center of ccREs are plotted in a ±2-kb range. (**B**) Significance of the overlaps between ncSTRs (heSTRs) and DHS from different tissues. ncSTRs were compared with bgSTRs, while heSTRs were compared with ncSTRs. (**C**) Significance of the overlaps between heSTRs and tissue-specific enhancers obtained from the human Tissue-specific Enhancer Database (TiED). Because of the scarcity of tissue-specific enhancers, a heSTR is considered to overlap with an enhancer if it is within a 10-kb distance to the enhancer. (**D**) Diagram illustrating ihTADs and TAD boundaries. (**E**) Distance from heSTRs (ncSTRs) to the nearest TAD boundaries. TAD boundaries identified in all seven tissues were used to compute the distance. (**F**) Significance of the overlaps between heSTRs and tissue-specific ihTADs. (**G**) Comparison of chromatin interaction frequency within ihTADs containing heSTRs versus those containing ncSTRs. *P* values were computed using the “wilcox.test” function in R and are shown on the right. (**H**) Significance of the overlaps between heSTRs and chromatin loops from different tissues. *P* values in (C), (F), and (H) were computed using the fisher.test function in R.

heSTRs, ncSTRs, and bgSTRs all exhibit enrichment toward the center of rDHS, PLS, pELS, and dELS but not for DNase-H3K4me3 and CTCF-only signatures, suggesting their association with gene expression regulation (Fig. 2A). Among these, ncSTRs display the strongest enrichment, indicating a robust link between evolutionary copy number conservation and regulatory elements. In contrast, heSTRs display weaker enrichment for PLSs and pELSs but greater enrichment for dELSs, suggesting a potentially more prominent role in distal regulation.

We investigated whether the regulatory association of STRs demonstrates tissue specificity. Using DHS peaks from 16 tissues [as defined by the ENCODE project (*38*)], we observed a significant enrichment of ncSTRs in brain DHSs compared to bgSTRs (OR = 1.32 and Fisher’s two-sided *P* = 3.52 × 10^−45^). heSTRs display an even higher enrichment in brain DHSs compared to ncSTRs (OR = 1.40 and *P* = 2.33 × 10^−7^) (Fig. 2B). This enrichment pattern remained consistent regardless of the presence of flanking TEs (fig. S6). Moreover, heSTRs display significant colocalization with brain-specific enhancers when compared to ncSTRs, as evidenced by tissue-specific enhancers obtained from the human Tissue-specific Enhancer Database (TiED; Fig. 2C and fig. S7) (*39*). These results suggest that heSTRs may play a role in brain-specific regulatory processes, particularly in distal regulations.

Chromatin three-dimensional (3D) interactions provide the structural basis for distal regulation. We therefore analyzed Hi-C data from seven tissues provided by the ENCODE project (table S3) (*38*). Our focus was on two enhancer-associated 3D structures (*40*, *41*): ihTADs (Fig. 2D) and chromatin loops. Using OnTAD (*40*), we identified between 6265 and 10,385 ihTADs across these tissues. In addition, leveraging annotations from the ENCODE project (*38*), we identified 1261 to 11,722 chromatin loops for these tissues (table S3). Previous studies reported that pathogenic STRs localize at TAD boundaries (*22*). Consistent with this, we found enrichment of both heSTRs (OR = 1.08 and Fisher’s two-sided *P* = 1.53 × 10^−3^) and ncSTRs (OR = 1.11 and *P* = 3.03 × 10^−39^) at TAD boundaries compared to bgSTRs (fig. S8A). Notably, these enrichment results were not influenced by differences in TE content among STR groups (fig. S8B). However, heSTRs were not more closely associated with boundaries than ncSTRs (Fig. 2E). Furthermore, we observed no tissue-specific patterns in ihTADs containing heSTRs (Fig. 2F), suggesting that heSTRs may not function by modifying TAD structures. In contrast, neuron-specific ihTADs with heSTRs show higher chromatin interaction frequencies (Fig. 2G), and heSTRs are enriched with chromatin loops in neurons (Fig. 2H). These findings underscore an association of heSTRs with neuron-specific long-range regulatory mechanisms.

### Association of genes potentially regulated by heSTRs with neuron-related functions and neurodevelopmental disorders

**Fig. 3.**
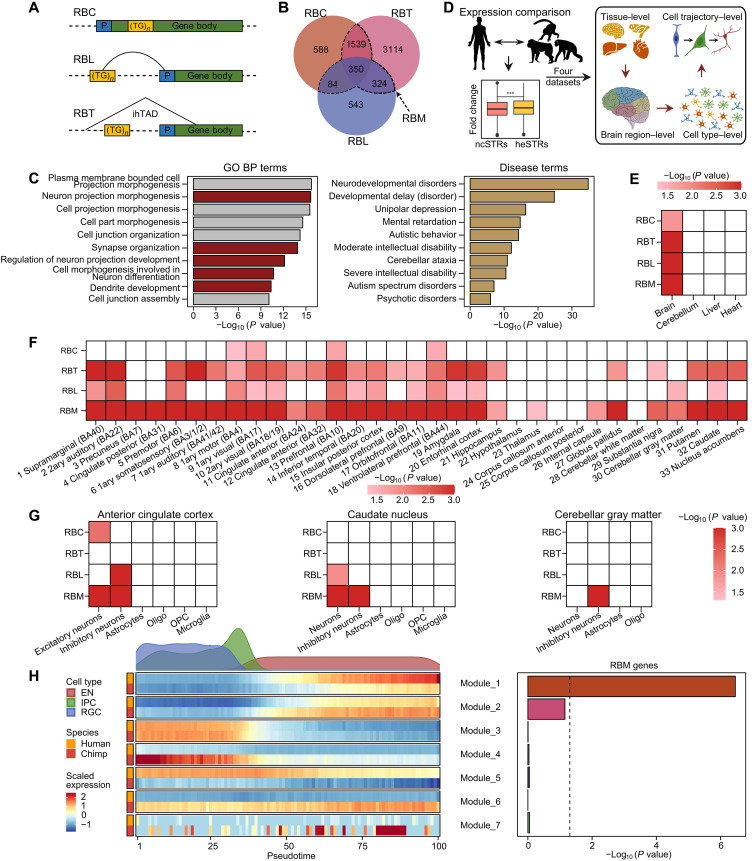
Cross-species expression data analyses for potential target genes regulated by heSTRs. (**A**) Definitions of three potential mechanisms for heSTR-involved regulation. P, promoter. (**B**) Venn diagram summarizing the number of potential target genes regulated by heSTRs. (**C**) Enrichment of Gene Ontology (GO) biological process (BP) terms and DisGeNET terms for RBM genes regulated by heSTRs. Regulation–by–multiple mechanisms genes (RBM genes) regulated by ncSTRs were used as the background for comparison. (**D**) Visualization of multiscale cross-species expression data analyses conducted in this study. Expression fold changes between human and NHP species were computed for each gene. Then, expression fold changes were compared between genes regulated by heSTRs and ncSTRs, and *P* values below the FDR-adjusted *P* value threshold of 0.1 are depicted in heatmaps (**E** to **G**). (E) Tissue-level expression enhancement for genes regulated by heSTRs between human and macaque. (F) Brain region–level expression enhancement for genes regulated by heSTRs between humans and NHPs. (G) Cell type–level expression enhancement for genes regulated by heSTRs between humans and NHPs in three different brain regions. 1ary, primary; 2ary, secondary; OPC, oligodendrocyte progenitor cells. (**H**) Development trajectory analysis of excitatory neuron lineage from brain organoid data in human and chimpanzee. The top panel of the left subfigure shows the density distribution of three cell types along the pseudotime. EN, excitatory neuron; IPC, intermediate progenitor cell; RGC, radial glial cell. Seven gene modules exhibiting consistent expression changes along the trajectory and between human and chimp are shown in the bottom panel of the left subfigure. Enrichment of RBM genes in these seven modules is shown in the right subfigure. *P* values are computed using the fisher.test function in R.

We next focused on identifying genes potentially regulated by heSTRs to better understand their possible phenotypic impacts. First, we identified 2561 genes housing heSTRs within their promoters or gene bodies, categorizing them as “regulation-by-colocalization genes” (RBC genes) (Fig. 3A). Because heSTRs are also linked to distal regulatory elements, we analyzed promoter-centered chromatin loops from a compendium spanning 27 tissues (*42*) from the Gene Expression Omnibus database (www.ncbi.nlm.nih.gov/geo; GSE86189). This analysis identified 1301 genes with loops overlapping at least two heSTRs, designated as “regulation-by-looping genes” (RBL genes) (Fig. 3A). In addition, given the association between heSTRs and TAD boundaries and the increased transcriptional activity often observed at these boundaries (*40*), we identified 5327 genes intersecting with ihTAD boundaries in seven tissues containing heSTRs, referred to as “regulation-by-TAD genes” (RBT genes) (Fig. 3A).

In total, we identified 6542 genes potentially influenced by heSTRs (Fig. 3B). To refine these categories, we identified genes unique to each regulation type—RBC-, RBL-, and RBT-only genes. Furthermore, we identified 2297 genes associated with at least two regulatory mechanisms, termed “regulation–by–multiple mechanisms genes” (RBM genes). These RBM genes likely represent a group more likely affected by heSTRs (Fig. 3B). Using the same approach, we identified genes potentially regulated by ncSTRs, resulting in four groups of background genes for comparison (fig. S9).

We conducted enrichment analysis on both Gene Ontology (GO) (*43*) biological process (BP) terms and DisGeNET (*44*) disease terms to compare genes regulated by heSTRs with their respective background genes. For GO enrichment, genes influenced by multiple mechanisms are significantly enriched in terms associated with neuronal development, such as “neuron projection morphogenesis,” “synapse organization,” and “regulation of neuron projection development” (Fig. 3C, left). Genes regulated by a single mechanism also exhibit enrichment in neuronal development terms, although with less statistical significance (fig. S10A).

Regarding disease terms, genes influenced by multiple mechanisms exhibit strong enrichment in those related to neurodevelopmental disorders (Fig. 3C, right). In contrast, the other categories of genes influenced by heSTR are enriched in a broader range of disease terms (fig. S10B). These results highlight the potential role of heSTRs in neuronal functions and neurodevelopmental disorders, suggesting their potential impact on human-specific phenotypes.

### Neuron-specific expression enhancement of genes potentially regulated by heSTRs

To validate the regulatory effects of heSTRs on their target genes, we gathered expression data from multiple species (*45*–*47*). This dataset included cross-species bulk RNA sequencing (RNA-seq) from various tissues, bulk and single-cell RNA-seq from different brain regions, and single-cell RNA-seq from brain organoids along their developmental trajectories (Fig. 3D and table S4). We then analyzed the expression fold change between humans and NHPs for genes regulated by heSTRs compared to those regulated by ncSTRs.

In a study by Cardoso-Moreira *et al.* (*45*), bulk RNA-seq data from various organs at different developmental stages were analyzed for representative animals, including humans and rhesus monkeys. The study highlighted greater transcriptional divergence between species and organs during late developmental stages (*45*). Focusing on four organs (brain, cerebellum, heart, and liver) sampled from both humans and rhesus monkeys during adulthood, we found that genes potentially regulated by heSTRs showed significant expression enhancement in the brain but not in the other three organs (Fig. 3E).

In a separate study, Khrameeva *et al.* (*46*) characterized the transcriptional profiles of 33 brain regions in humans, gorillas, bonobos, and rhesus monkeys. Using these data, we found that genes most strongly affected by heSTRs (RBM genes) exhibit significant expression enhancement in humans compared to the three NHP species across most brain regions (Fig. 3F). Genes regulated solely by TADs (RBT-only genes) show a similar expression pattern, though in fewer brain regions. In contrast, genes regulated only by colocalization (RBC-only genes), which are affected by heSTRs in promoters or gene bodies, exhibit the weakest expression enhancement. These findings support the regulatory role of heSTRs in enhancing gene expression in the human brain.

Khrameeva *et al.* (*46*) also provided single-nucleus RNA-seq data from brain regions such as the anterior cingulate cortex, caudate nucleus, and cerebellar gray matter, allowing us to examine the cell type–specific expression impact of heSTRs. Genes strongly influenced by heSTRs (RBM genes) show significant expression enhancement in nearly all types of neuronal cells across these brain regions. In contrast, genes regulated by looping (RBL-only) or colocalization (RBC-only) mechanisms exhibit expression enhancement in only one or two brain regions (Fig. 3G), highlighting the neuron-specific regulation by heSTRs.

In addition, using a single-cell expression atlas of nonbrain organs in cynomolgus monkeys (*48*) and corresponding human organ data (*49*), we found that RBM genes are not up-regulated in any cell types in nonbrain organs. However, the other three classes of heSTR-regulated genes show some up-regulation in immune cell types (fig. S11). This further confirms the neuron-specific regulatory impact of heSTRs.

In another study, Kanton *et al.* (*47*) analyzed brain organoid expression data from humans and chimpanzees, using cross-species pseudotime alignment of neuronal differentiation trajectories at the single-cell level. Because inhibitory neurons were incompletely differentiated in the brain organoid data (*47*), we focused on the developmental trajectory of excitatory neurons. Through time-series clustering analysis, we identified seven gene modules with consistent expression patterns along the trajectory in both humans and chimpanzees. Among these, one module enriched with RBM genes showed increased expression along the neuronal developmental trajectory in both species, with a stronger expression in humans (Fig. 3H). No modules were enriched with the other three classes of heSTR-regulated genes (fig. S12A). Genes in this module are associated with functions promoting excitatory neuron development (fig. S12B), suggesting that heSTRs regulate target genes during the later stages of excitatory neuron development.

**Fig. 4.**
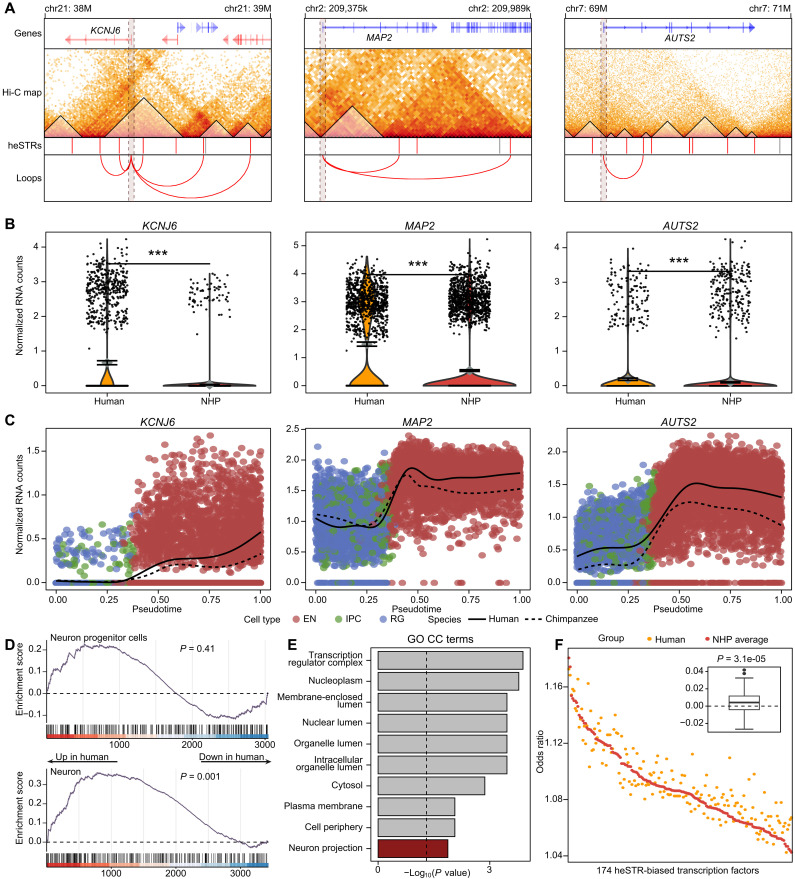
Examples of target genes regulated by heSTRs and the potential mechanisms of regulatory impacts. (**A**) Illustrations of three potential target genes (*KCNJ6*, *MAP2*, and *AUTS2*) regulated by heSTRs, showing transcript structure (top row), motor neuron Hi-C heatmaps at a resolution of 10 kb displaying ihTADs (second row), heSTR locations (third row; red bars for heSTRs that regulate the target gene and gray bars for other heSTRs), and promoter-capture loops intersecting with heSTRs (bottom row). The dashed shaded box indicates the promoter region. chr21, chromosome 21. (**B**) Expression comparison between human and chimpanzee for *KCNJ6*, *MAP2*, and *AUTS2* in anterior cingulate cortex excitatory neurons. (**C**) Expression comparison between human and chimpanzee for *KCNJ6*, *MAP2*, and *AUTS2* during excitatory neuron development. (**D**) Assay for Transposase-Accessible Chromatin (ATAC) peak enrichment analysis comparing heSTRs versus non-heSTRs in neuron progenitor cells and neuron. For peaks overlapping with ncSTRs, human/chimp fold changes were ranked in descending order and then compared with ranks of peaks overlapping heSTRs. *P* values calculated using the “gene set enrichment analysis” function from clusterProfiler (*75*). (**E**) GO cellular component enrichment analysis for 174 transcription factors (TFs) showing preferential binding to heSTR-flanking regions. CC, cellular component. (**F**) Comparison of the ORs of TF binding sites between humans and NHPs for 174 heSTR-biased TFs. The wilcox.test function in R was used to determine whether the difference between human ORs and the mean ORs of NHPs is significantly greater than 0.

We selected three genes to illustrate the regulatory impact of heSTRs. *KCNJ6*, which encodes a potassium ion channel protein that modulates dopaminergic neuron excitability, is associated with Keppen-Lubinsky syndrome (*50*). *MAP2*, encoding microtubule-associated protein 2, serves as a crucial regulator of the neuronal dendritic cytoskeleton and is implicated in various neurological disorders (*51*). *AUTS2*, encoding a subunit of the Polycomb Repressive Complex 1-like complex, is associated with neural development and identified as a candidate risk gene for autism (*52*). *KCNJ6* is subject to potential regulation by seven heSTRs via looping and TAD mechanisms, while *MAP2* and *AUTS2* are potentially regulated by heSTRs through looping and TAD mechanisms, respectively (Fig. 4A). All three genes exhibit robust expression enhancement in human excitatory neurons within the anterior cingulate cortex brain region (Fig. 4B). Furthermore, analysis of organoid data confirms their enhanced expression pattern along the neuronal developmental trajectory (Fig. 4C). These instances highlight that heSTRs may influence the expression of genes critical for the development of neuron cells.

### Association of heSTRs with neuron-specific enhanced chromatin accessibility and increased transcription factor binding sites

The association of heSTRs with gene expression enhancement in neuron cells raises the question of how these elements exert their regulatory roles. Increased gene expression is often linked to greater chromatin accessibility in the regulatory regions that regulate the gene. Thus, it is reasonable to investigate the association of heSTRs with chromatin accessibility. The brain organoid study conducted by Kanton *et al.* (*47*) also provided single-cell chromatin accessibility data for neuron cells and neuron progenitor cells in both humans and chimpanzees. Using this dataset, we identified Assay for Transposase-Accessible Chromatin (ATAC) peaks overlapping with heSTRs and ncSTRs and found no significant difference in peak lengths between these two groups (Wilcoxon’s two-sided *P* = 0.637; fig. S13). We then calculated the fold change in read counts between humans and chimpanzees for ATAC peaks associated with heSTRs and ncSTRs, ranking the peaks by their fold changes. Compared to ncSTR-associated ATAC peaks, those associated with heSTRs exhibit significantly enhanced accessibility in neuron cells but not in neuron progenitor cells (Fig. 4D). This supports our hypothesis and provides strong evidence for the neuron cell–specific regulatory impact of heSTRs.

Increased chromatin accessibility can enhance the binding of transcription factors (TFs), thereby boosting the expression of target genes. For heSTRs, greater chromatin accessibility may specifically promote the binding of neuron-related TFs, leading to an increase in the expression of genes important for neuronal functions. To test this hypothesis, we systematically examined all potential TF binding sites within the 500-bp flanking heSTRs and ncSTRs in the hg38 genome sequence (details in Materials and Methods). We then calculated the ratio of binding sites in heSTRs to those in ncSTRs for each TF and identified 174 TFs that preferentially bind to heSTRs [OR > 1 and false discovery rate (FDR)–adjusted *P* < 0.05]. Notably, these 174 TFs are enriched for the GO term “neuron projection,” suggesting their relevance to neuron-related functions (Fig. 4E). For example, Retinoic Acid Receptor Gamma, the most notable TF, belongs to the retinoic acid receptor family and is crucial for differentiation and neurogenesis (*53*). Another important TF, Orthodenticle Homeobox 2, plays a key role in the fate determination of neuron progenitors (*54*).

We then inspected whether these heSTR-biased TFs exhibit increased binding site availability in humans compared to NHPs. To do this, we calculated the ORs of the number of binding sites of heSTRs to ncSTRs for each of the 174 TFs based on the reference genome sequences of seven NHP species and averaged the results. Comparing the OR of these 174 TFs between humans and NHPs revealed significantly higher ORs in humans (Fig. 4F), while no such significance was found for randomly selected TFs (fig. S14). These findings suggest that heSTRs may induce neuron-specific regulatory effects by increasing the availability of binding sites for neuron-related TFs in their flanking regions.

### Association of heSTRs with population-level functional STRs and pathogenic STRs

**Fig. 5.**
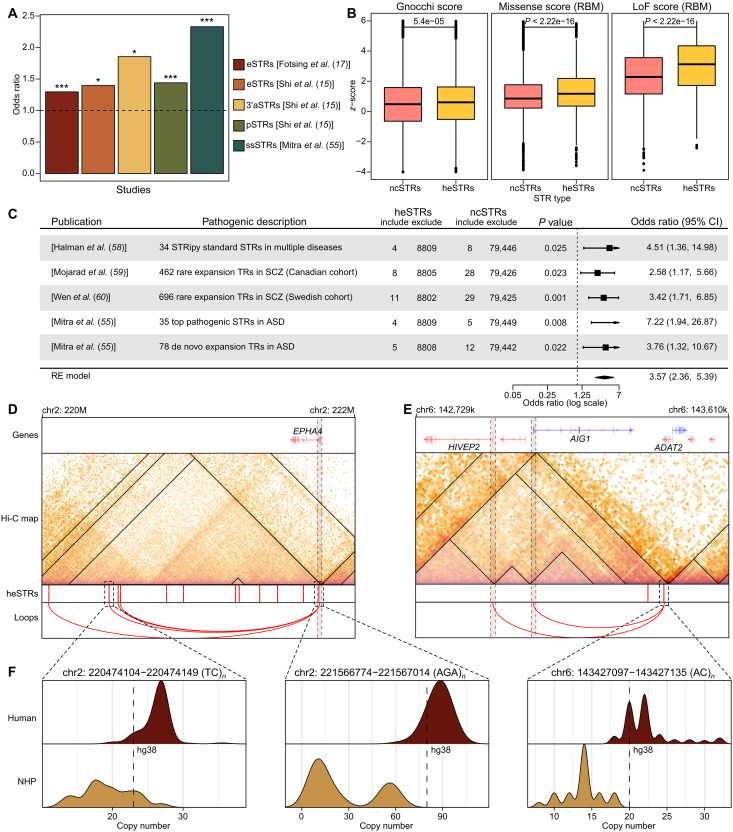
The association of heSTRs with population-level functional STRs and pathogenic STRs. (**A**) Significance of the overlaps between heSTRs and functional STRs identified from population studies. *P < 0.05 and ***P < 0.001 (Fisher’s exact test). (**B**) Comparison of genomic constraint scores between heSTR and ncSTR. Gnocchi, missense, and loss-of-function (LoF) scores are collected from gnomAD version 3 and version 2, respectively. (**C**) Significance of the overlaps between heSTR and pathogenic STRs reported by five datasets. CI, confidence interval; ASD, autism spectrum disorder; SCZ, schizophrenia; RE model, random effect model. (**D** and **E**) Examples of pathogenic STRs whose genomic loci correspond to heSTRs, highlighted in the dashed box. (**F**) Copy number distributions of the pathogenic heSTRs in (D) and (E) in human and NHP populations.

In this study, our focus was on identifying STRs with significant copy number expansions in humans. While our primary focus was on cross-species comparisons, other studies have explored the diversity of STRs within the human population. For example, Shi *et al.* (*15*) identified a subset of STRs called population highly variable STRs (pSTRs), which show considerable variability across human superpopulations, particularly in relation to nervous system functions. Shi *et al.* (*15*) also identified expression-associated STRs (eSTRs) and 3′ untranslated region (3′UTR) alternative polyadenylation STRs (3′aSTRs), displaying copy number polymorphisms linked to gene expressions and distal polyadenylate site selection, respectively. In addition, Fotsing *et al.* (*17*) also reported on eSTRs. Here, we examined the relationship between heSTRs and the functional STRs described in these studies. Compared to ncSTRs, heSTRs exhibit greater copy number variability within the human population (fig. S15A). Consistent with this, heSTRs demonstrate a significant enrichment in pSTRs, eSTRs, and 3′aSTRs (Fig. 5A), suggesting that a substantial proportion of heSTRs may contribute to phenotypic diversity within the human population.

We also examined whether heSTRs are subject to selective pressure within the human population. The SISTR tool (*55*) identifies STRs that are constrained by selection through population genetic simulations. The SISTR study identified 6901 STRs under strong negative selection (referred to as ssSTRs) from a total of 62,941 STRs with 2- to 4-bp motifs (*55*). Compared to non-ssSTRs, ssSTRs exhibit significantly lower variability in copy numbers (fig. S15B). When examining heSTRs and ncSTRs that overlap with the STRs analyzed by SISTR, we found that heSTRs are significantly enriched for ssSTRs across all motif lengths (fig. S15C). This suggests that heSTRs may be under stronger mutational constraints than ncSTRs, indicating that a subset of heSTRs is subject to heightened evolutionary pressures within the human population.

Population-level data from the gnomAD database further support the idea of selective constraints on heSTRs. Analysis of genomic constraint scores (Gnocchi scores) from gnomAD (version 3) (*56*) revealed stronger selective pressures on regions containing heSTRs compared to those with ncSTRs (Fig. 5B). In addition, gene intolerance scores from gnomAD (version 2) (*57*) indicate that genes potentially regulated by heSTRs experience greater selective pressure than those regulated by ncSTRs (Fig. 5B and fig. S16). These findings suggest that, although heSTRs generally exhibit greater variability in the human population, a subset is likely under stronger selective constraints, highlighting their functional importance.

In addition to population-level variation, STRs with abnormal copy numbers have been linked to various diseases, as documented by the STRipy database (*58*). We analyzed 34 pathogenic STRs from this database, focusing on the standard repeat types and excluding those not represented in our reference panel, such as VNTRs, imperfect GCN repeats, and nested repeat types. Four of these pathogenic STRs overlap with heSTRs, showing a higher representation compared to ncSTRs (Fig. 5C). Moreover, recent whole-genome sequencing studies on schizophrenia and autism (*55*, *59*, *60*) have identified tandem repeats with rare or de novo copy number expansions associated with these conditions. When compared to ncSTRs, heSTRs exhibit a significant enrichment in the pathogenic STRs reported in these studies (Fig. 5C).

Most pathogenic STRs in the STRipy database are genic (33 genic versus 1 nongenic) (fig. S17A). In contrast, ~40% of pathogenic STRs identified in recent studies on schizophrenia and autism are nongenic (fig. S17A). To determine whether the association between heSTRs and pathogenic STRs is influenced by the distribution of genic and nongenic types, we repeated the pathogenic STR enrichment analysis separately for genic and nongenic STRs. Notably, the proportion of nongenic STRs among both heSTRs and ncSTRs is similar to that observed in pathogenic STRs from schizophrenia and autism studies, with no notable differences between the groups (fig. S17A). In both genic and nongenic categories, heSTRs remained enriched for pathogenic STRs compared to ncSTRs (fig. S17, B and C), although some individual intersections lacked statistical significance due to a lower count of pathogenic STRs. When all pathogenic STRs were considered together, substantial overlaps with heSTRs were observed in both genic and nongenic regions (fig. S17D). These findings confirm that the enrichment of heSTRs for pathogenic STRs is independent of genomic annotation.

The identification of target genes potentially regulated by heSTRs in this study provides insights into the mechanism by which these repeats contribute to diseases. For example, two rare expanded TRs in the schizophrenia cohort were identified as heSTRs. Analysis of the target genes potentially regulated by these two heSTRs revealed *EPHA4*, a gene essential for neuronal migration (*61*) and potentially linked to depressive behavior (*62*). This gene may be regulated through looping and colocalization mechanisms (Fig. 5D). Similarly, in the autism cohort, a de novo rare expanded TR was identified as a heSTR within the 3′UTR of *ADAT2*. This heSTR may regulate *AIG1* and *HIVEP2* through the TAD structure mechanism (Fig. 5E). Notably, *HIVEP2* is a highly confident autism risk gene according to the Simons Foundation Autism Research Initiative database (*63*). All three heSTRs show pronounced copy number expansion in humans compared to NHPs (Fig. 5F). These examples not only show a strong association between heSTRs and diseases but also highlight the importance of distal regulation in understanding the phenotypic impact of pathogenic STRs.

## DISCUSSION

Previous studies have identified various forms of accelerated evolution in the human genome, such as HARs (*7*–*11*) and HAQERs (*12*), associating them with neuronal-related phenotypes. However, the role of copy number expansion events for STRs in human phenotypic evolution has not been well explored. In this study, we used a large number of haploid genomes assembled from third-generation sequencing, with a representative evolutionary background that includes both closely and distantly related NHP species. We identified 8813 heSTRs. The substantial overlap of heSTRs with previously identified loci under accelerated evolution further supports their involvement in this process. Through a multidimensional analysis incorporating 3D genomic structural features and cross-species expression and regulatory data, we investigated the impact of heSTRs on human neuronal phenotypes. We found a strong association of heSTRs with neuron-specific regulatory features, including neuron-specific regulatory signals, chromatin loops, and ihTADs specifically implicated in neuronal function. In addition, genes potentially regulated by heSTRs were found to enrich functions related to neuronal development and exhibit enhanced expression in human neuronal cells. We also observed that heSTRs notably overlap with pathogenic STR loci identified in schizophrenia and autism cohorts. These findings highlight heSTRs as a previously overlooked class of neuron-specific regulatory elements and suggest their potential contribution to human-specific phenotypes during evolution.

The inclusion of three evolutionarily distant NHP species in the background is crucial for the robust identification of genuine heSTRs. To demonstrate this, we repeated the computational pipeline for identifying heSTRs while excluding these three distant NHPs from the evolutionary backgrounds. The resulting heSTRs exhibit a weaker association with brain-specific DHSs, and the DHSs overlapping with the newly identified “heSTRs” display no tissue specificity (fig. S18), underscoring the necessity of constructing a more representative evolutionary background. Consistent with this, the study by Bi *et al.* (*10*) also suggested that incorporating genomes from 49 primate species improves the identification of reliable LinARs. As more distant primate haplotype genomes become available, we anticipate further enhancement in our ability to identify heSTRs.

A notable finding in our investigation of heSTRs is their pronounced association with neuron-specific distal regulatory signals. A recent study has proposed that STRs may influence gene expression through chromatin loops (*64*), further supporting the potential roles of heSTRs in distal regulations. In addition, previous research has shown that pathogenic STRs are often located near TAD boundaries (*22*), suggesting a possible mechanism for their involvement in distal regulation. Our analysis revealed that both heSTRs and ncSTRs are enriched at TAD boundaries compared to genome-wide STRs. This suggests that colocalization with TAD boundaries may be a common feature of functionally important STRs. However, as heSTRs are not preferentially located at TAD boundaries compared to ncSTRs, colocalization with TAD boundaries is not specific to STRs undergoing HSE.

Because of the association of heSTRs with distal regulation, we expanded the scope of potential target genes of heSTRs beyond those found in gene promoters or gene bodies (referred to as RBC genes), which are typically analyzed for phenotypic impacts. In this study, we also considered genes regulated by chromatin loops (RBL genes) and by innermost TADs (RBT genes). Among the target genes of heSTRs, those regulated by multiple mechanisms (RBM genes) show the highest enrichment with neuronal development–related GO terms and neuronal disorders. These genes also exhibit the most pronounced expression enhancement in neuron cells. In contrast, genes regulated solely by heSTRs in their promoters or gene bodies (RBC-only genes) display much weaker associations with neuron-related functions and expression. This highlights the importance of understanding the expression impact of STRs in the context of distal regulations. Previous studies (*15*, *17*, *18*) on eSTRs typically focus on identifying STR-gene pairs with linear correlations in one or multiple tissue expression conditions. Our study suggests that a joint analysis of the effects of multiple STRs in the context of distal regulations may facilitate understanding the tissue-specific regulatory effects of STRs on gene expression.

In this study, we have not only shown neuron-specific expression enhancement for genes potentially regulated by heSTRs but also provided evidence for the association of heSTRs with neuron-specific enhanced chromatin accessibility and increased TF binding sites, offering insights into the likely mechanisms on how heSTRs alter gene expression. On the basis of these findings, we propose that heSTRs may enhance gene expression by increasing chromatin accessibility and influencing TF binding in human neuron cells. Horton *et al.* (*65*) recently demonstrated that STRs substantially enhance TF binding affinity with flanking TF motif sequences, which partially explains how heSTRs interact with TFs. Influencing the TF binding process may be a general mechanism for the phenotypic impacts of accelerated evolution events. For example, HARs have been shown to function as neural enhancers (*7*–*9*), potentially altering TF binding affinity through mutations in motif sequences. While these studies primarily focus on changes in TF motif sequences, STRs may also influence the TF binding process independently of specific motifs. Future research into accelerated evolution may benefit from considering both STR expansion and motif sequence changes.

While our study provides valuable insights into the evolution of STRs, it is limited to STRs annotated as “Simple_repeat” by RepeatMasker (*33*) in the hg38 reference genome. This constraint, common to other genome-wide STR studies (*15*, *16*), excludes potentially relevant STRs, particularly those within TEs or complex genomic regions. These excluded STRs may not only exhibit copy number expansion but also show sequence variations or more complex genomic alterations. Furthermore, although third-generation sequencing–based haploid genomes currently represent the optimal approach for STR copy number quantification (*66*), the genomes in our collection were constructed using diverse sequencing platforms and assembly methods (table S1), which may potentially affect the accuracy of STR copy number quantification (*67*, *68*). As genome-wide STR annotations improve and more high-quality haploid genomes become available, future studies will likely identify additional human-specific STRs, leading to a more comprehensive understanding of accelerated STR evolution in the human genome.

This study provides a valuable resource for prioritizing pathogenic STR variants and exploring potential disease-causing mechanisms. The identified heSTRs exhibit noteworthy associations with loci of known pathogenic STRs and likely pathogenic STRs identified from cohorts of neurodevelopmental disorders. Given the frequent observation of STR copy number variation in neurological disorders (*20*), the 8813 heSTRs could help prioritize pathogenic STR variants identified in a given cohort. In addition, the potential target genes identified in this study can be used to formulate hypotheses regarding disease-causing mechanisms, which can then be tested by experiment. As demonstrated by the tool PrimateAI-3D (*69*), the combination of machine learning with evolutionary background constraints can effectively distinguish between benign mutations and pathogenic mutations in coding regions. We expect that as more sites of accelerated evolution, such as heSTRs, are found, evolutionary constraints will improve the ability to identify the pathogenic variants in noncoding regions.

## MATERIALS AND METHODS

### Data collection and STR genotyping

We obtained human genome sequences assembled using third-generation sequencing from the Human Genome Structural Variation Consortium (HGSVC; https://ftp.1000genomes.ebi.ac.uk/vol1/ftp/data_collections/HGSVC2/release/v1.0/assemblies/) and Human Pangenome Reference Consortium (HPRC; www.ncbi.nlm.nih.gov/bioproject/730822) projects, comprising genome sequences from 35 and 43 individuals, respectively. To ensure that the resulting haploid genomes were unrelated, we excluded the genome sequences of three children from parent-child trios in the HGSVC dataset. In addition, we omitted the genome sequence of HG002 from the HPRC dataset due to duplication with NA24385 in the HGSVC dataset. In total, we obtained 148 haploid human genomes. For details regarding the ethnicity of the individuals corresponding to these haploid genomes, please refer to table S1. Furthermore, we obtained 26 haploid NHP genomes assembled using third-generation sequencing from the National Center for Biotechnology Information genome datasets (www.ncbi.nlm.nih.gov/genome/).

For STR genotyping, we initially constructed the reference STR panel by retrieving the track named “RepeatMasker” for the hg38 assembly from the University of California, Santa Cruz (UCSC) genome browser (https://genome.ucsc.edu/cgi-bin/hgTrackUi?g=rmsk). We selected loci classified as Simple_repeat with motif lengths ranging from 1 to 6 bp, resulting in 670,429 STRs. Subsequently, we used Minimap2 (*34*) to map each STR, along with the 500-bp flanking sequence, to each haploid genome. Default parameters (-ax asm5) were used for humans, while parameters -ax asm10 were used for NHPs to accommodate higher divergence. We identified the longest mapped region in a haploid genome as the mapped region for the corresponding reference STR and applied RepeatMasker (v.4.1.4) (*33*) to this region to verify the presence of the STR, using parameters -s and -noint to enhance detection sensitivity. However, there were instances where more than one STR was detected. In such cases, we selected the STR with boundaries closest to a distance of 500 bp from the boundary of the mapped region. Last, we computed the copy number of the STR by dividing its length by the motif length reported by RepeatMasker (v.4.1.4) (*33*).

To validate our STR genotyping approach, we compared our pipeline with vamos (*66*) and tandem-genotypes (*70*). Focusing on the 114,947 STRs that overlap with vamos’s predefined TR panel (vamos.effMotifs-0.1.GRCh38) from the 160,054 homologous STRs used to derive our ncSTRs and heSTRs, our pipeline showed high concordance with both methods in copy numbers identified in human genomes (Spearman correlation > 0.96) (fig. S19, A to C). In NHPs, the correlation between these methods all decreased with evolutionary distance (fig. S19, D to F), although vamos and tandem-genotypes showed higher similarity to each other due to the use of the same alignment coordinates provided by us. For motif prediction, our pipeline achieved about 96% concordance with vamos’s prediction (fig. S19G). While vamos and tandem-genotypes are well benchmarked for human genomes, there is currently no gold standard for STR quantification in NHPs. Given RepeatMasker’s established use in tandem repeat evolution study (*28*), we retained our pipeline for NHPs.

### Identification of ncSTRs and heSTRs

Starting from potential homologous STRs, we filtered out STRs detected in fewer than 10 human haploid genomes to ensure an adequate number of observations. To identify ncSTRs and heSTRs, we used a series of statistical tests on these remaining STRs. First, we used the “diptest” function from the Python diptest module to filter out STRs whose copy numbers in evolutionarily close NHPs exhibited complex distributions or violated the assumption of unimodal distribution (FDR-adjusted *P* < 0.05). Subsequently, we used the “ks_2samp” function from the Python scipy.stats module to identify STRs whose copy numbers in evolutionarily distant NHPs significantly (FDR-adjusted *P* < 0.05) deviated from the distribution observed in evolutionarily close NHPs. The remaining STRs were classified as ncSTRs.

Next, for each ncSTR, we used the “ranksums” function from the Python scipy.stats module to identify those with significantly (Bonferroni-adjusted *P* < 0.01, one-tailed test) greater copy numbers in humans compared to NHPs. These STRs were designated as heSTRs.

### Genomic characteristic profiling of heSTRs

To analyze the genomic distribution of STRs, we obtained gene annotations from the “ncbiRefSeq” track on the UCSC genome browser (*71*). We then used the BEDTools (*72*) “annotate” function to categorize STRs into different genomic regions. Specifically, we defined the promoter region as 3 kb upstream and downstream of the transcription start site. Any STRs that did not intersect with any promoters or gene bodies were assigned to the intergenic region.

To investigate the association of STRs with regulatory features, we obtained six types of ccREs based on the reference genome of hg38 from the ENCODE project (www.encodeproject.org/; accessions provided in table S3). The distance of STRs to ccREs was computed using the BEDTools “closest” function.

For analyzing the association of heSTRs with DHSs, we acquired DHS peak annotations from 16 tissues from the ENCODE project (accessions provided in table S3). We then used the BEDTools “intersect” function to identify DHS peaks intersecting with heSTRs. To assess whether heSTRs exhibit any tissue preference compared to ncSTRs, we used the “fisher.test” function in R.

Regarding the association of STRs with tissue-specific enhancers, we obtained tissue-specific enhancers from the TiED (http://lcbb.swjtu.edu.cn/TiED) (*39*). The genome coordinates of tissue-specific enhancers were converted from hg19 to hg38 using LiftOver (*73*). In addition, the TiED enhancers were extended into 10- to 40-kb flanked segments using the BEDTools “slop” function to enhance the intersection with STRs.

We obtained Hi-C data in hic format for seven representative tissues from the ENCODE project (accessions provided in table S3). Subsequently, we used the “hicConvertFormat” function of HiCExplorer (*74*) with default parameters to generate chromatin interaction matrices at a 10-kb resolution. These matrices were then processed using OnTAD (*40*) to identify ihTADs, using recommended parameters (-penalty 0.1 and -maxsz 200). In addition, chromatin loop annotations for these Hi-C datasets were downloaded from the ENCODE project (accessions provided in table S3).

### Enrichment analysis of heSTR target genes

GO enrichment analysis was conducted using the “enrichGO” function of the clusterProfiler R package (*75*), using the GO database from 1 July 2022 (*43*). In addition, DisGeNET (*44*) enrichment analysis was performed using the “disease_enrichment” function of the disgenet2r R package (*44*).

### Cross-species expression data analysis

Detailed accessions and download links for the cross-species expression data (*45*–*49*) analyzed in this study are provided in table S4. We used different methods for bulk RNA-seq data and single-cell RNA-seq data. For bulk RNA-seq data, we used the “exactTest” function from the edgeR (*76*) R package with default parameters. For single-cell RNA-seq data, we used the “FindMarkers” function of the Seurat (*77*) R package, with the “test.use” parameter set to “wilcox” and the “logfc.threshold” set to 0.

For brain organoid cross-species expression data analysis, we initially divided single cells into 100 bins based on their pseudotime provided by Kanton *et al.* (*47*). Subsequently, we calculated the average expression of each gene in human (or chimpanzee) cells within a bin to represent the gene’s expression in that bin. Next, we applied the maSigPro (*78*) R package to identify gene modules with consistent expression patterns across species and along the 100 pseudotime bins. When using maSigPro, we set the “rsq” parameter in the “get.siggenes” function to 0.1, the “cluster.method” parameter to “hclust”, and “k” in the “see.genes” function to 7, resulting in seven gene modules.

### Cross-species single-cell ATAC-seq data analysis

We retrieved the BAM files of brain organoid single-cell ATAC sequencing (ATAC-seq) from ArrayExpress (www.ebi.ac.uk/biostudies/arrayexpress) using accession codes E-MTAB-8089 and E-MTAB-8043. These BAM files were then converted into FASTQ files using the BEDTools “bamtofastq” function. Next, we used Bowtie2 (*79*) to align these data to the hg38 and panTro6 reference genomes and identified ATAC peaks using the “callpeak” function in MACS2 (*80*). To ensure cross-species compatibility, we used LiftOver (*73*) to convert the genome coordinates of peaks from panTro6 to hg38 (more than 95% sequence similarity requirement), enabling the identification of ATAC peaks present in both human and chimpanzee genomes. Subsequently, featureCount (*81*) was used to count reads falling within specific ATAC peaks. The BEDTools intersect function was then applied to identify ATAC peaks intersecting with STRs.

To compute the fold changes of peak accessibility, we used the FindMarkers function of the R package Seurat. For this analysis, we set the parameters logfc.threshold to 0 and test.use to “LR,” which is recommended for ATAC-seq data analysis.

### TF binding site predictions

We retrieved the binding motifs of 775 TFs in MEME format from the JASPAR database (*82*). The reference genomes of human and NHP species, including hg38, panTro6, panPan3, gorGor6, ponAbe3, nomLeu3, rheMac10, and macFas5, were downloaded from the UCSC genome browser (https://hgdownload.soe.ucsc.edu/goldenPath). For each ncSTR (including heSTR), we obtained the 500-bp flanking sequences of an STR in hg38 and identified the corresponding sequences in reference NHP sequences using LiftOver (*73*). Subsequently, we used FIMO (v.5.4.4) (*83*) with default parameters to scan for potential transcription factor binding sites within the flanking regions of an STR for all TFs.
