# Supplementary material for: Association of human-specific expanded short tandem repeats with neuron-specific regulatory features
Source: Sci Adv. 2025 May 30;11(22):eadp9707. doi: 10.1126/sciadv.adp9707 (PMC12124357; doi:10.1126/sciadv.adp9707)
Supplement: Supplementary file 1 — Figs. S1 to S19 Legends for tables S1 to S4 [file sciadv.adp9707_sm.pdf]

Science Advances
AAAS

Supplementary Materials for

Association of human-specific expanded short tandem repeats with neuron-specific regulatory features

Qiming Liu and Weidong Tian

Corresponding author: Weidong Tian, weidong.tian@fudan.edu.cn

Sci. Adv. **11**, eadp9707 (2025)
DOI: 10.1126/sciadv.adp9707

The PDF file includes:

Figs. S1 to S19
Legends for tables S1 to S4

Other Supplementary Material for this manuscript includes the following:

Tables S1 to S4

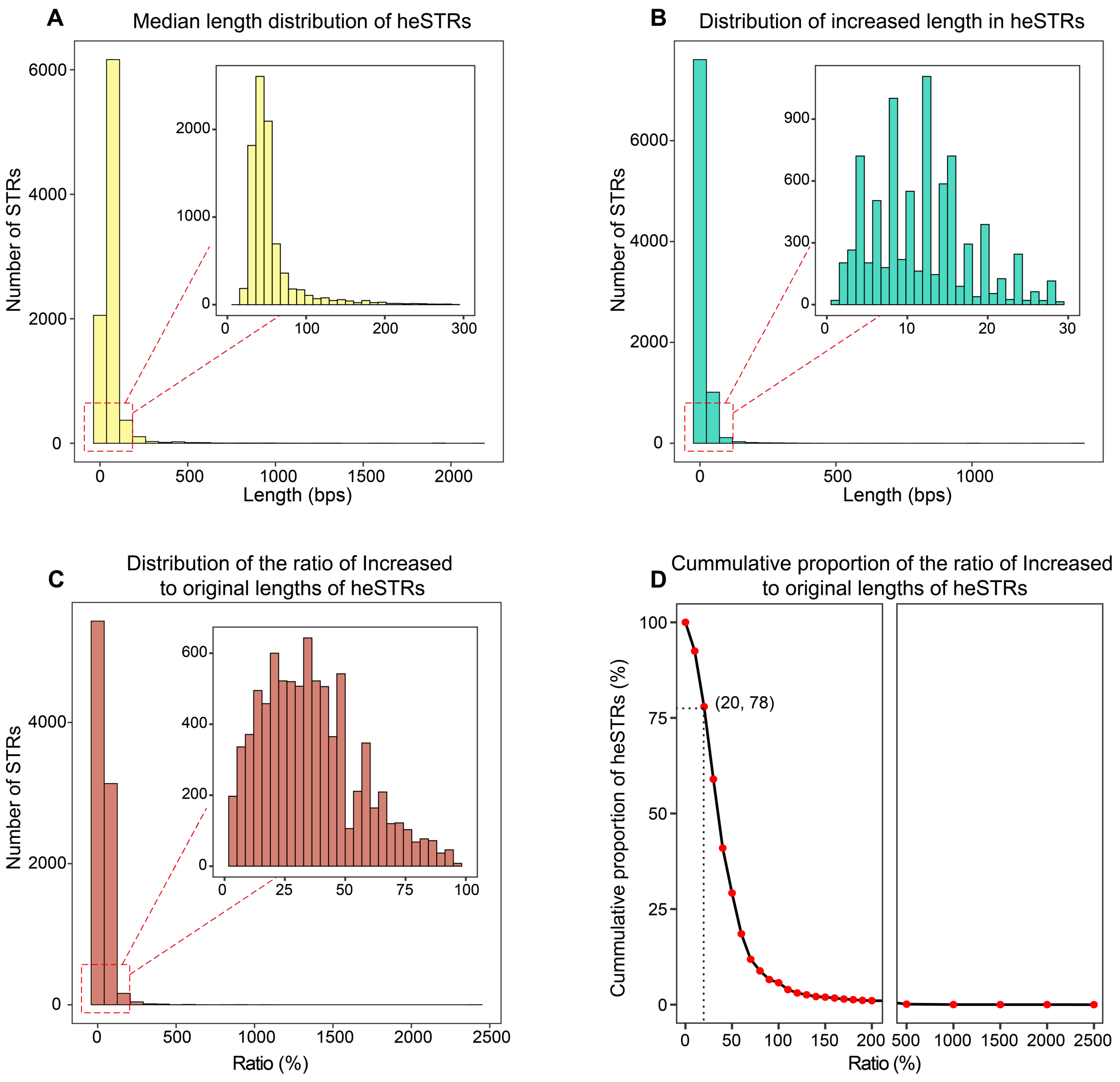

Fig. S1.

Length characteristics of heSTRs. (A), Distribution of the median lengths of heSTRs. (B), Distribution of the increase in median length of heSTRs. (C), Distribution of the ratio of increased to original lengths of heSTRs. (D), The cumulative curve of heSTR expansion ratios.

A

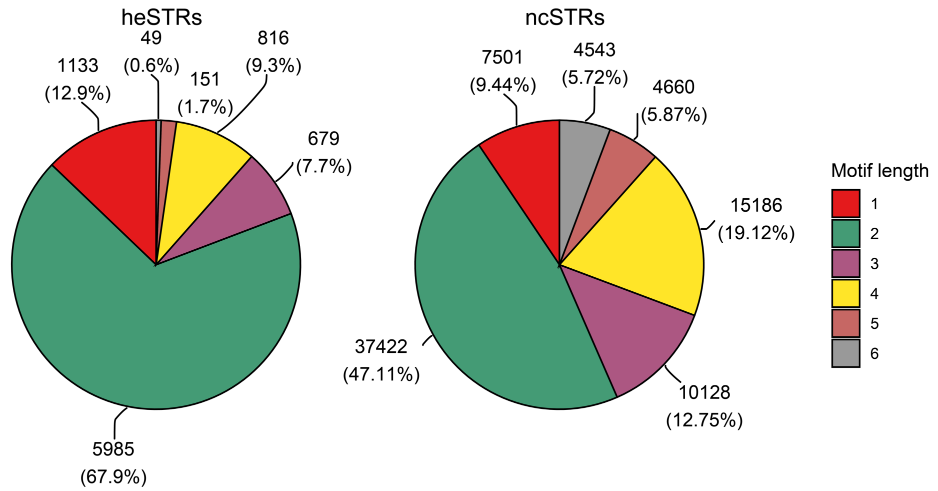

B

Motif component enrichment of heSTRs

Motif	heSTRs Yes	heSTRs No	ncSTRs Yes	ncSTRs No	OR	p−value	q−value
AC	4638	4084	25526	46598	2.07 [1.98, 2.17]	2.53e−223	9.35e−222
A	1131	7591	7472	64652	1.29 [1.2, 1.38]	3.62e−13	1.68e−12
AT	812	7910	7956	64168	0.83 [0.77, 0.89]	7.03e−07	1.37e−06
AG	534	8188	3886	68238	1.15 [1.04, 1.26]	4.83e−03	5.42e−03
AAT	335	8387	3823	68301	0.71 [0.63, 0.8]	1.55e−09	4.10e−09
AAAT	250	8472	2986	69138	0.68 [0.6, 0.78]	2.41e−09	5.96e−09
AGAT	148	8574	2020	70104	0.6 [0.5, 0.71]	1.89e−10	6.35e−10
AAC	123	8599	1311	70813	0.77 [0.64, 0.93]	5.94e−03	6.46e−03
AAAC	100	8622	1233	70891	0.67 [0.54, 0.82]	4.97e−05	7.08e−05
ACCT	68	8654	984	71140	0.57 [0.44, 0.73]	1.45e−06	2.68e−06
AAGT	66	8656	2610	69514	0.2 [0.16, 0.26]	6.31e−62	1.17e−60
CCG	60	8662	968	71156	0.51 [0.39, 0.66]	3.92e−08	8.54e−08
ACT	56	8666	1254	70870	0.37 [0.27, 0.48]	1.50e−17	1.11e−16
AAAAT	52	8670	772	71352	0.55 [0.41, 0.74]	1.00e−05	1.62e−05
AAAG	45	8677	944	71180	0.39 [0.28, 0.53]	2.16e−12	8.88e−12
AGG	36	8686	1020	71104	0.29 [0.2, 0.4]	8.16e−19	7.55e−18
ACAT	35	8687	752	71372	0.38 [0.26, 0.54]	2.12e−10	6.53e−10
AAAAC	35	8687	927	71197	0.31 [0.21, 0.43]	3.74e−16	2.31e−15
AAGG	29	8693	971	71153	0.24 [0.16, 0.35]	1.02e−20	1.26e−19
ACC	28	8694	602	71522	0.38 [0.25, 0.56]	1.50e−08	3.47e−08
ACG	27	8695	773	71351	0.29 [0.19, 0.42]	1.15e−14	6.05e−14
AATT	20	8702	422	71702	0.39 [0.24, 0.61]	3.32e−06	5.85e−06
AGGG	16	8706	515	71609	0.26 [0.14, 0.42]	3.44e−11	1.27e−10
AAG	14	8708	376	71748	0.31 [0.17, 0.52]	2.85e−07	5.86e−07
AATAT	8	8714	242	71882	0.27 [0.12, 0.55]	1.43e−05	2.21e−05
ACAG	6	8716	201	71923	0.25 [0.09, 0.55]	4.19e−05	6.20e−05
ACGGG	6	8716	87	72037	0.57 [0.2, 1.29]	2.39e−01	2.39e−01
AACT	5	8717	207	71917	0.2 [0.06, 0.47]	6.35e−06	1.07e−05
ATAT	5	8717	163	71961	0.25 [0.08, 0.6]	2.69e−04	3.31e−04
AGGGG	5	8717	158	71966	0.26 [0.08, 0.62]	5.34e−04	6.37e−04
ACCGCC	5	8717	19	72105	2.18 [0.64, 6.03]	1.74e−01	1.79e−01
ACAC	4	8718	127	71997	0.26 [0.07, 0.68]	1.76e−03	2.04e−03
CCCG	4	8718	157	71967	0.21 [0.06, 0.55]	1.72e−04	2.27e−04
AAATT	4	8718	276	71848	0.12 [0.03, 0.31]	1.54e−09	4.10e−09
AACAT	4	8718	72	72052	0.46 [0.12, 1.23]	1.39e−01	1.47e−01
CCCCG	4	8718	151	71973	0.22 [0.06, 0.57]	2.31e−04	2.95e−04
AATATT	4	8718	161	71963	0.21 [0.06, 0.54]	1.22e−04	1.68e−04

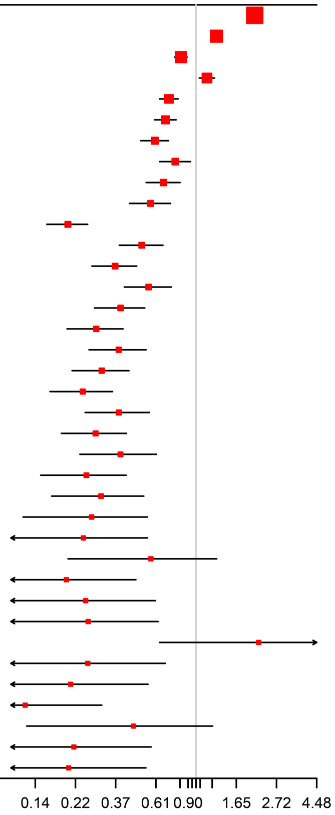

Fig. S2.

Motif composition and enrichment analysis of heSTRs versus ncSTRs. (A), Distribution of motif lengths between heSTRs and ncSTRs. (B), Enrichment analysis of specific motif types in heSTRs compared to ncSTRs. Only motifs occurring ≥4 times in heSTRs were included in the analysis. P-values were calculated using Fisher's exact test. Error bars represent 95% confidence intervals.

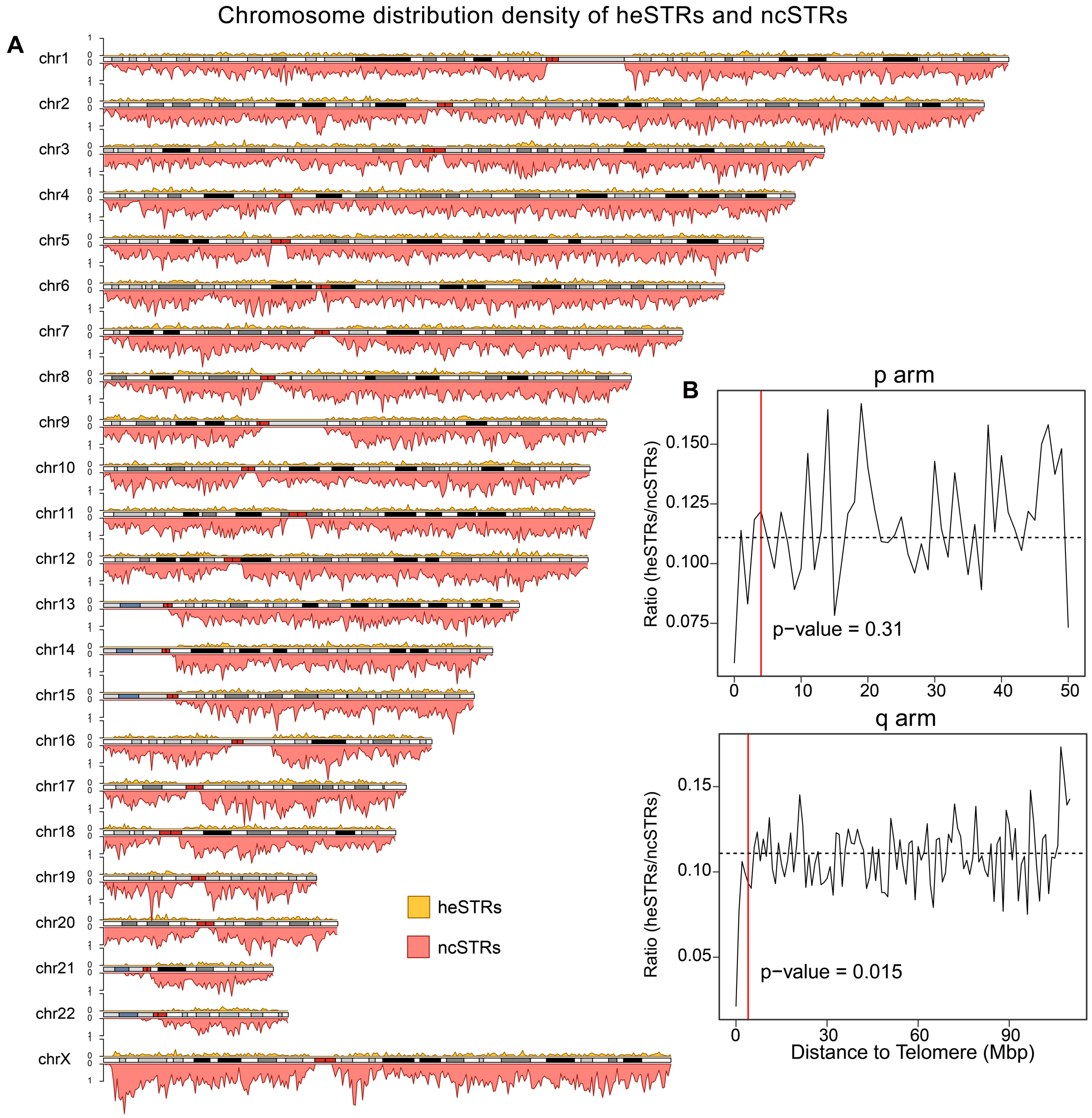

Fig. S3.

Genome-wide distribution of heSTRs. (A), Distribution landscape of heSTRs and ncSTRs across each chromosome. (B), Ratio of the number of heSTRs to ncSTRs in the human genome, averaged over 1-Mbp intervals across chromosomes. The red line indicates the position 5 Mbp from the telomere, and the dotted line represents the expected ratio of heSTRs. P-values were computed using `wilcox.test` function in R.

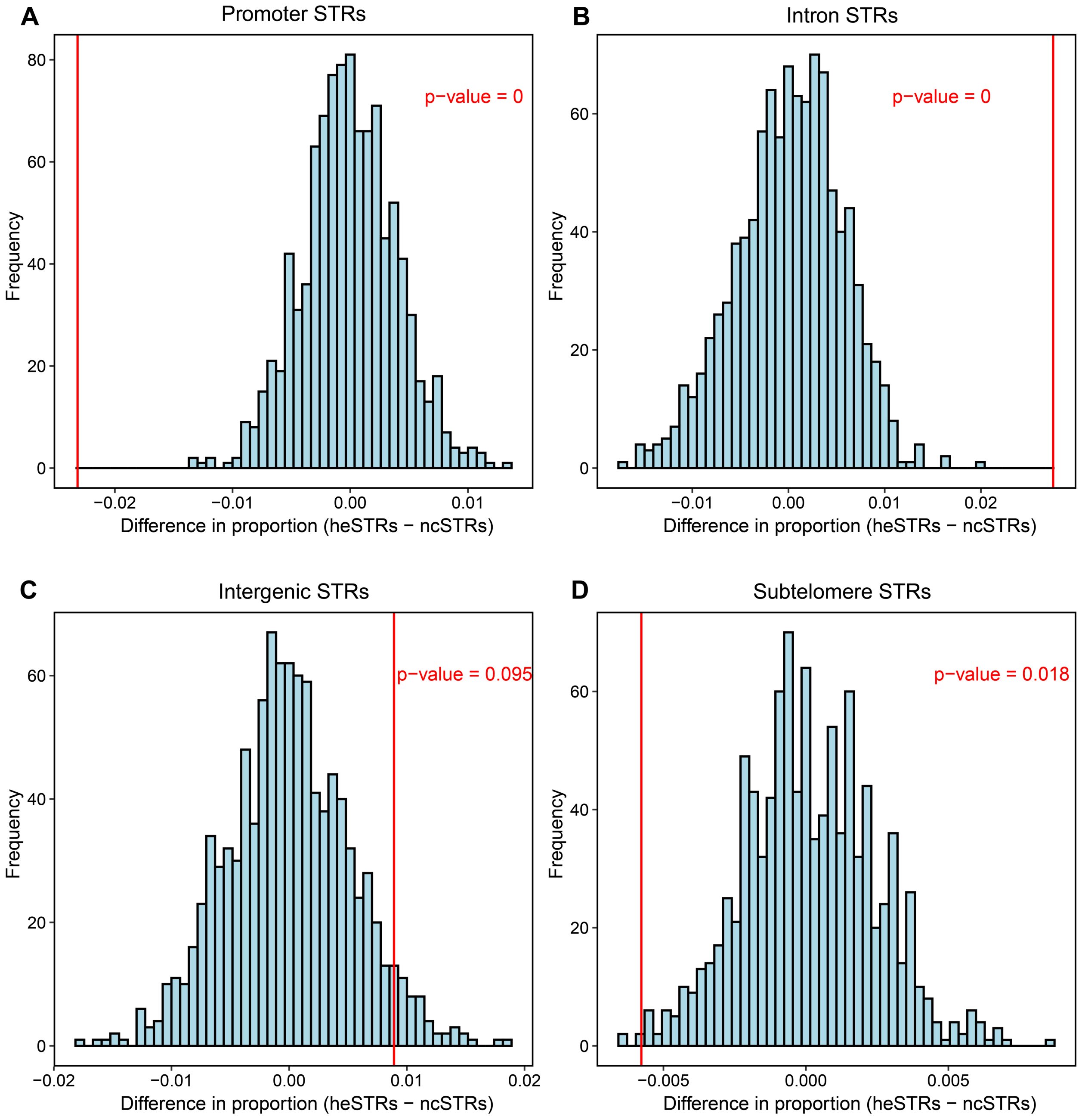

Fig. S4.

Genomic distribution of heSTRs and ncSTRs analyzed by permutation tests. (A-D), Comparison of heSTR and ncSTR proportions across different genomic regions: (A) promoter regions, (B) intronic regions, (C) intergenic regions, and (D) subtelomeric regions. Red vertical lines indicate the observed differences in proportions between heSTRs and ncSTRs. The histograms show the distribution of differences obtained from 1,000 random permutations. P-values were derived from these permutation tests.

A

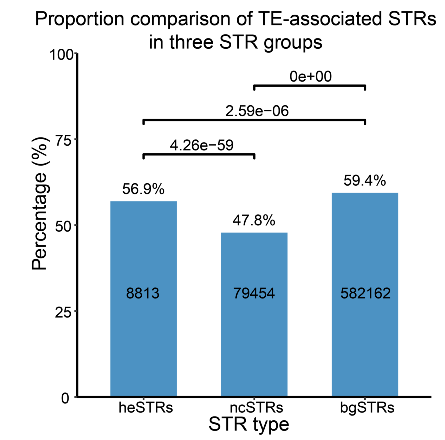

B

Enrichment of TE Families in the Flanking Regions of heSTRs

repFamily	repClass	heSTRs Yes	heSTRs No	ncSTRs Yes	ncSTRs No	OR	p−value	q−value
TcMar−Mariner	DNA	22	7814	173	53048	0.86 [0.53, 1.35]	5.92e−01	8.07e−01
TcMar−Tigger	DNA	237	7599	1516	51705	1.06 [0.92, 1.22]	4.05e−01	6.85e−01
hAT−Blackjack	DNA	25	7811	170	53051	1 [0.63, 1.52]	1.00e+00	1.00e+00
hAT−Charlie	DNA	323	7513	2542	50679	0.85 [0.76, 0.96]	8.53e−03	2.56e−02
hAT−Tip100	DNA	57	7779	407	52814	0.95 [0.7, 1.25]	7.81e−01	8.36e−01
CR1	LINE	77	7759	698	52523	0.74 [0.58, 0.94]	1.29e−02	3.24e−02
L1	LINE	2314	5522	13075	40146	1.28 [1.22, 1.35]	4.75e−20	3.57e−19
L2	LINE	649	7187	5789	47432	0.74 [0.68, 0.8]	4.02e−13	2.01e−12
RTE−X	LINE	20	7816	151	53070	0.9 [0.53, 1.44]	7.32e−01	8.36e−01
ERV1	LTR	201	7635	1287	51934	1.06 [0.91, 1.23]	4.56e−01	6.85e−01
ERVL	LTR	540	7296	3340	49881	1.1 [1, 1.21]	4.47e−02	9.58e−02
ERVL−MaLR	LTR	481	7355	3311	49910	0.98 [0.89, 1.09]	7.45e−01	8.36e−01
Gypsy	LTR	28	7808	161	53060	1.18 [0.76, 1.77]	4.45e−01	6.85e−01
Alu	SINE	2152	5684	13440	39781	1.12 [1.06, 1.18]	5.99e−05	2.25e−04
MIR	SINE	646	7190	6563	46658	0.64 [0.58, 0.69]	4.14e−28	6.21e−27

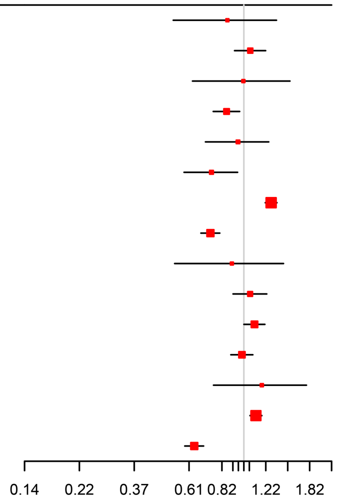

Fig. S5.

Association of heSTRs with transposable elements (TEs). (A), Proportion of TE-associated STRs in bgSTRs, ncSTRs, and heSTRs. TE-associated STRs were defined as those with transposable elements within their 100-bp flanking regions. P-values were calculated using `prop.test` function in R. (B), Enrichment of TE families within the flanking regions of heSTRs compared to ncSTRs. Only TE families with more than 10 occurrences in heSTRs were evaluated. TE families marked in red indicate statistically significant enrichment after FDR correction. P-values were computed using `fisher.test` function in R.

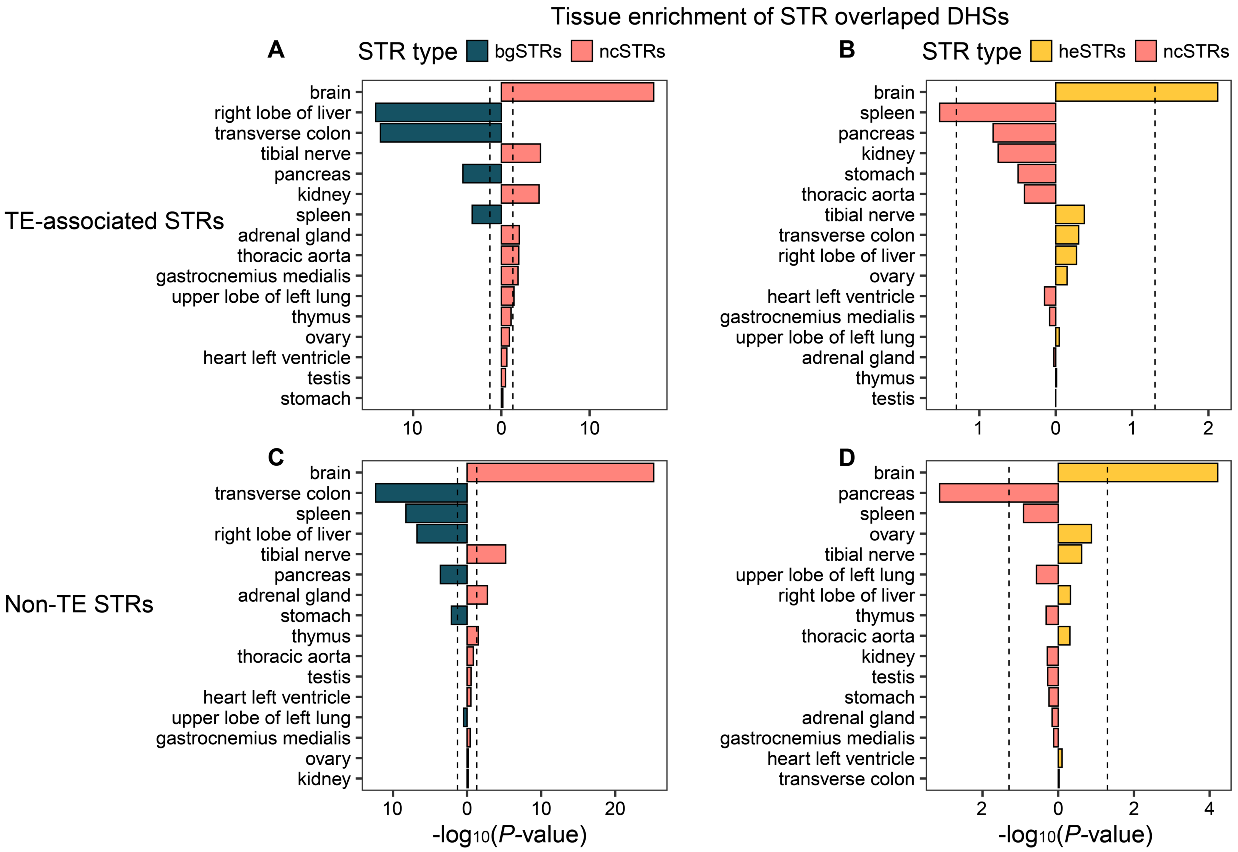

Fig. S6.

Differential DHS enrichment analysis between TE-associated and non-TE STRs. (A-D), DHS enrichment analysis comparing (A) ncSTRs versus bgSTRs and (B) heSTRs versus ncSTRs in TE-associated STRs; (C) ncSTRs versus bgSTRs and (D) heSTRs versus ncSTRs in non-TE STRs. The dashed lines represent the significance threshold (P = 0.05). P-values in (A-D) were determined using Fisher's exact test.

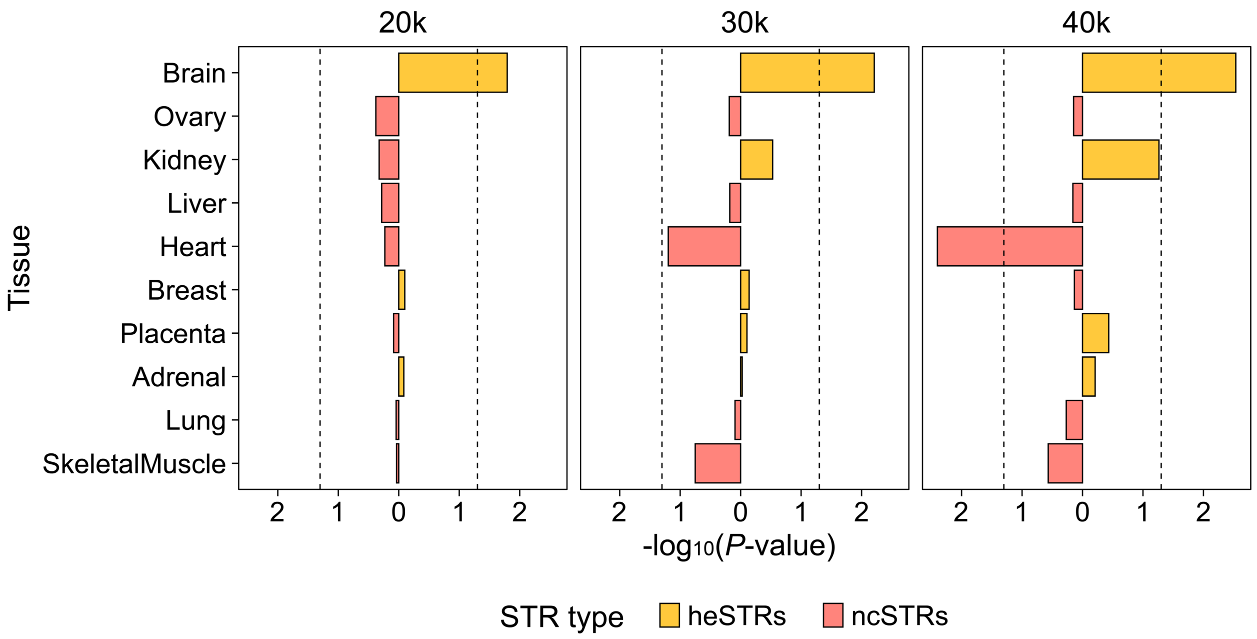

Fig. S7.

Enrichment analysis between heSTRs and tissue-specific enhancers. This figure shows the significance of the overlaps between heSTRs and tissue-specific enhancers obtained from the TiED database, with the co-localization distance set to 20k, 30k, or 40k bp.

A

Enrichment of heSTRs and ncSTRs in TAD boundary regions

Group<br>Target vs background	Target Yes	Target No	Background Yes	Background No	OR	p−value
heSTRs vs ncSTRs	2901	5912	26735	52719	0.97 [0.92, 1.01]	1.72e−01
heSTRs vs bgSTRs	2901	5912	182404	399758	1.08 [1.03, 1.12]	1.53e−03
ncSTRs vs bgSTRs	26735	52719	182404	399758	1.11 [1.09, 1.13]	3.03e−39

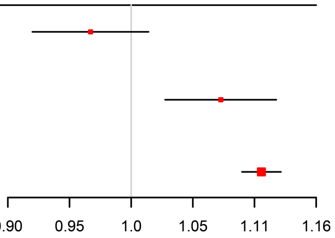

B

	Group<br>Target vs background	Target Yes	Target No	Background Yes	Background No	OR	p−value
TE-associated STRs	heSTRs vs ncSTRs	1579	3438	12190	25808	0.97 [0.91, 1.04]	3.94e−01
	heSTRs vs bgSTRs	1579	3438	105933	239935	1.04 [0.98, 1.1]	2.00e−01
	ncSTRs vs bgSTRs	12190	25808	105933	239935	1.07 [1.05, 1.09]	6.54e−09
Non-TE STRs	heSTRs vs ncSTRs	1322	2474	14545	26911	0.99 [0.92, 1.06]	7.63e−01
	heSTRs vs bgSTRs	1322	2474	76471	159823	1.12 [1.04, 1.2]	1.38e−03
	ncSTRs vs bgSTRs	14545	26911	76471	159823	1.13 [1.11, 1.15]	2.18e−27

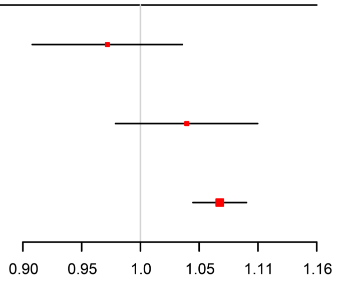

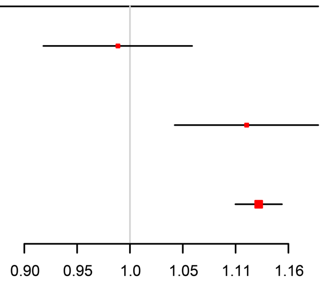

Fig. S8.

Enrichment analysis of STRs in TAD boundary regions. (A), Enrichment analysis comparing the distribution of heSTRs, ncSTRs and bgSTRs in TAD boundary regions. (B), Separate enrichment analyses for TE-associated STRs (top) and non-TE STRs (bottom). TAD boundary regions were defined as ±10 kb from the annotated boundaries based on Hi-C data resolution. The odds ratios and P-values were calculated using Fisher's exact test.

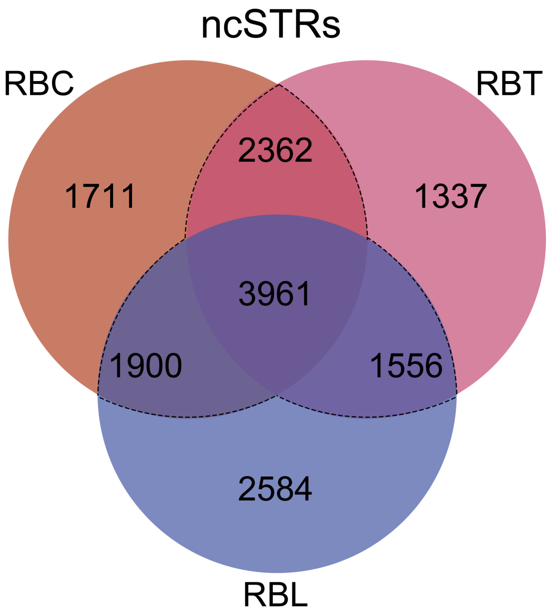

Fig. S9.

Distribution and overlap of genes regulated by ncSTRs through different regulatory mechanisms. Venn diagram summarizing the number of potential target genes regulated by ncSTRs, revealing 9,934 RBC-genes, 10,001 RBL-genes, and 9,216 RBT-genes. A total of 9,779 genes were classified as RBM-genes.

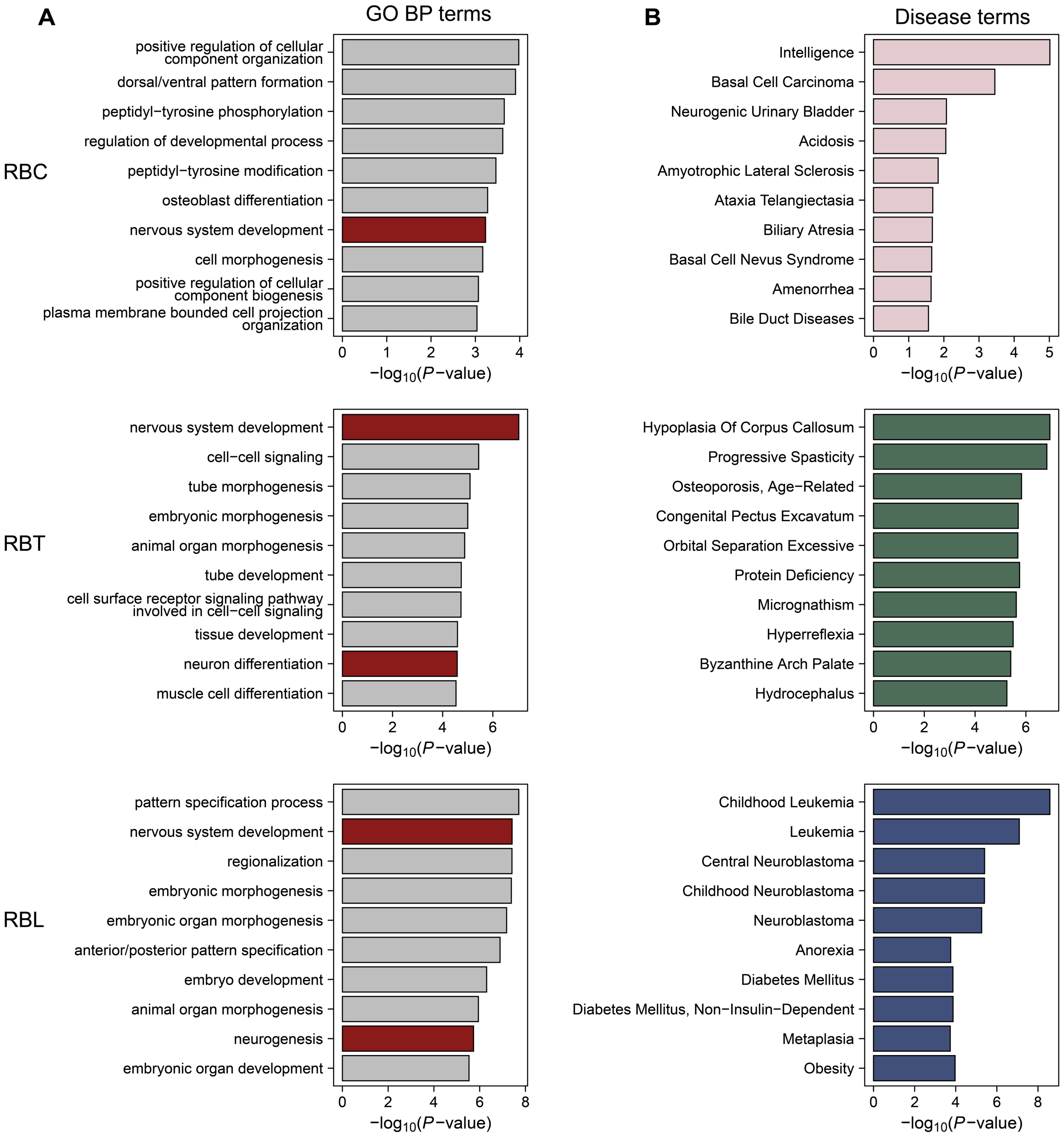

Fig. S10.

Functional and disease enrichment analysis of genes uniquely regulated by heSTRs through single regulatory mechanisms. Enrichment of GO BP terms (A) and DisGeNET terms (B) for genes exclusively regulated by heSTRs through one mechanism type (i.e., RBC, RBT, and RBL-only genes).

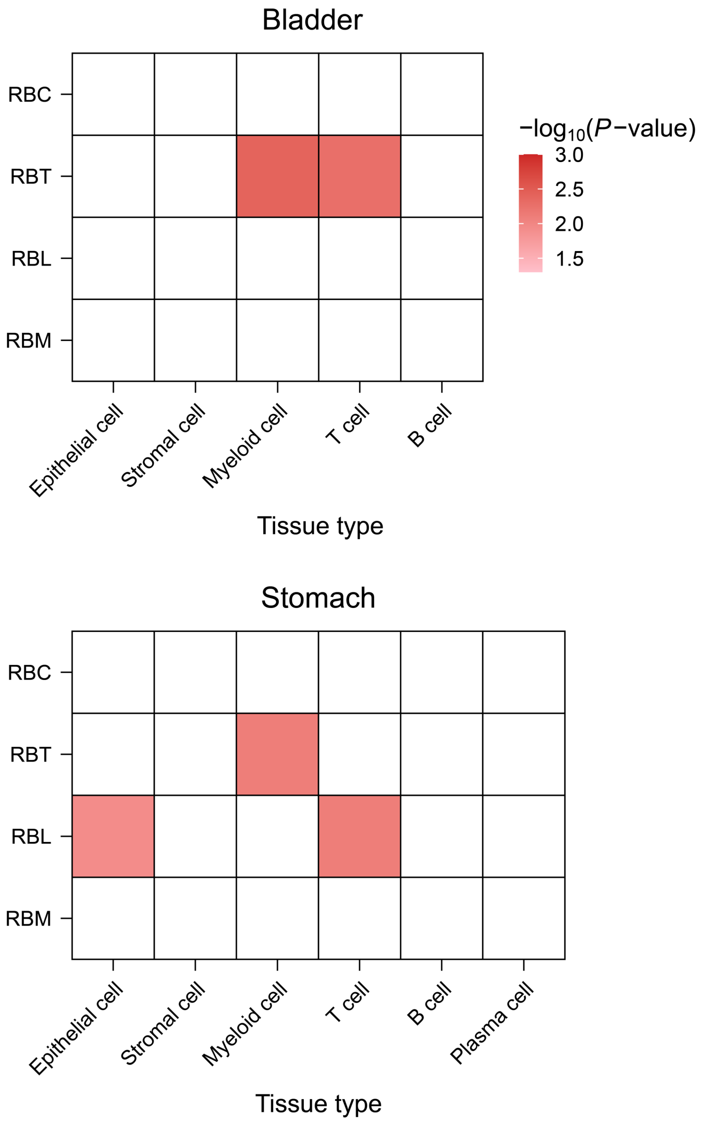

Fig. S11.

Cell type-level expression enhancement for genes regulated by heSTRs between human and cynomolgus in bladder and stomach. Cell type labeling consistency across datasets was ensured using the Seurat function `TransferData` with default parameters on homologous genes to transfer cell type annotations from cynomolgus to human. The comparison was performed on eight organs, with bladder and stomach exhibiting significant expression enhancement and shown here.

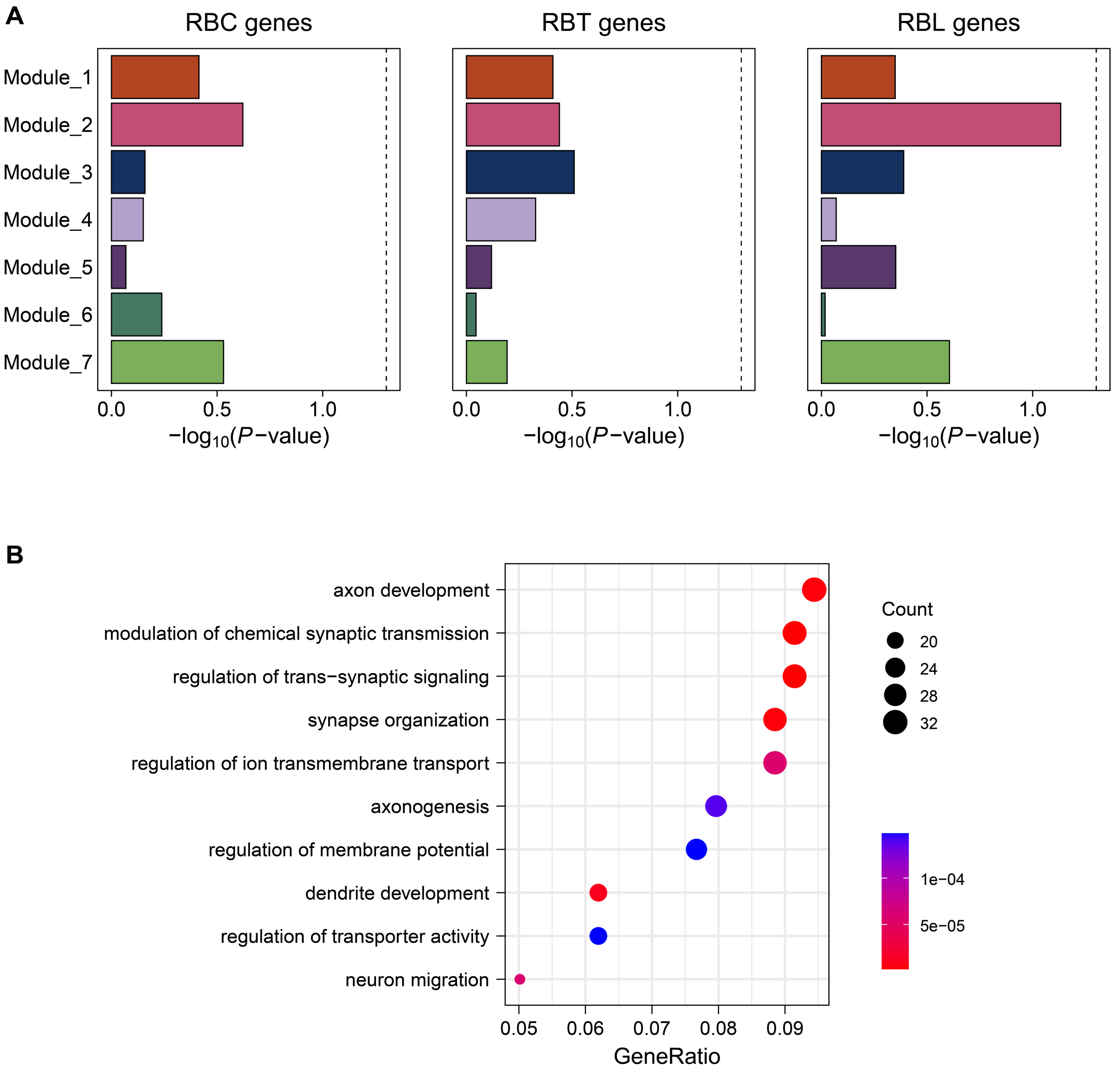

Fig. S12.

Association analysis between heSTR target genes and pseudotime differential gene modules. (A), Enrichment of genes regulated by heSTRs through only one mechanism type (i.e., RBC, RBT, and RBL-only genes) in these seven modules. (B), Enrichment of GO BP terms for genes in "module 1".

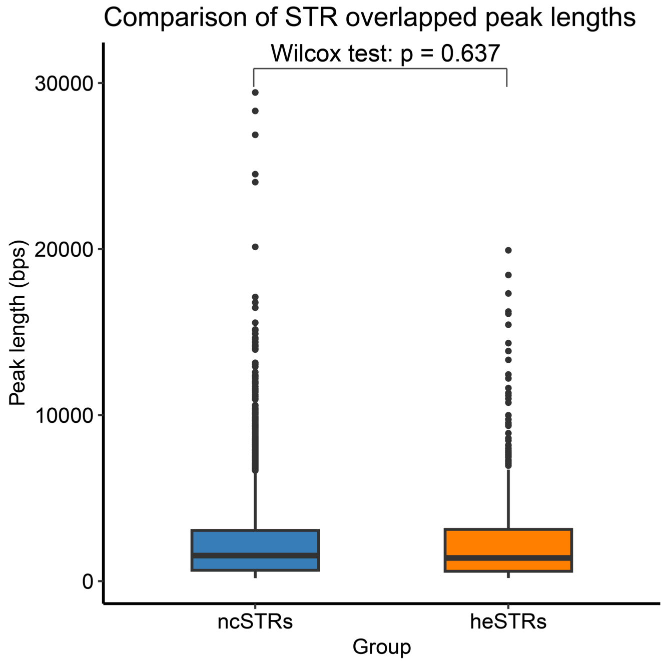

Fig. S13.

Comparison of peak lengths between ATAC-seq peaks overlapping with ncSTRs versus heSTRs. P-value was calculated using two-sided Wilcoxon rank-sum test.

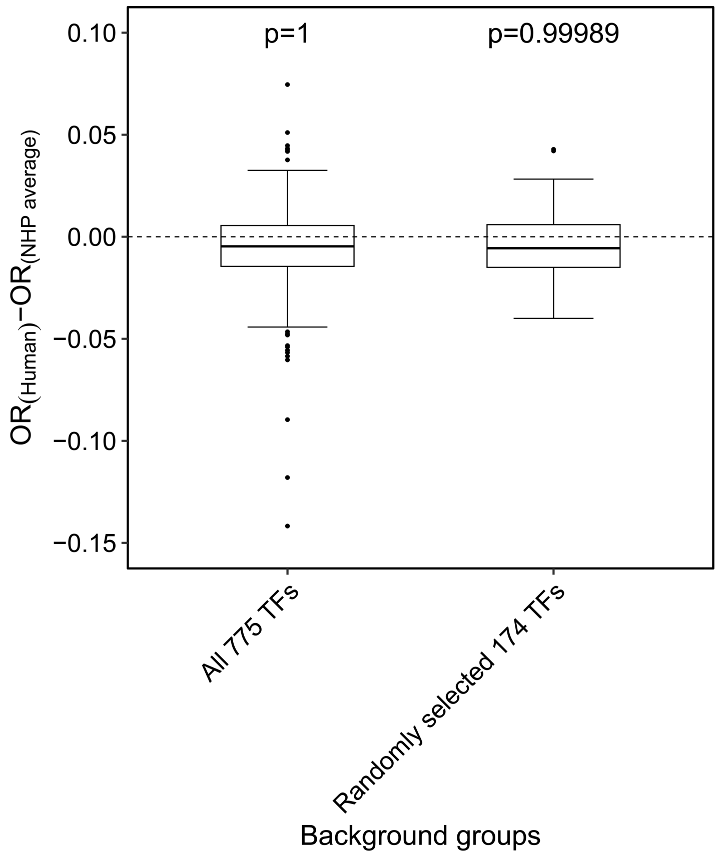

Fig. S14.

Comparison of the odds ratios of TFs binding sites between human and NHPs for background TFs. This figure shows the differences between the odds ratio of the number of binding sites in humans and the corresponding average in NHPs for all 775 TFs and a randomly selected set of 174 TFs.

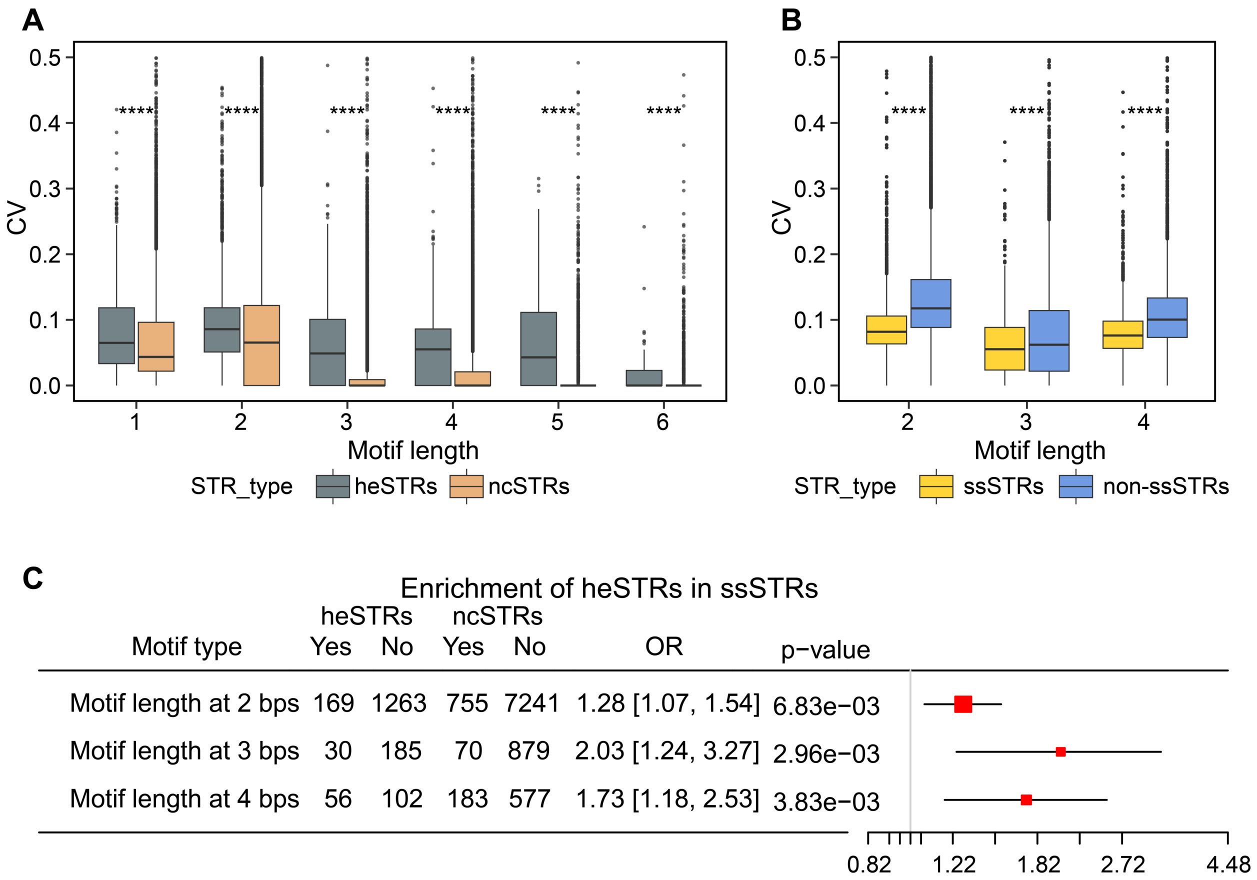

Fig. S15.

Copy number variations of heSTRs and ssSTRs at different motif lengths within the human population. (A), Coefficient of variation (CV) in copy numbers for heSTRs and ncSTRs within the human population at different motif lengths. (B), CV in copy numbers for ssSTRs and non-ssSTRs within the human population at different motif lengths. (C), Enrichment of ssSTRs in heSTRs compared to ncSTRs. P-values were computed using `fisher.test` function in R.

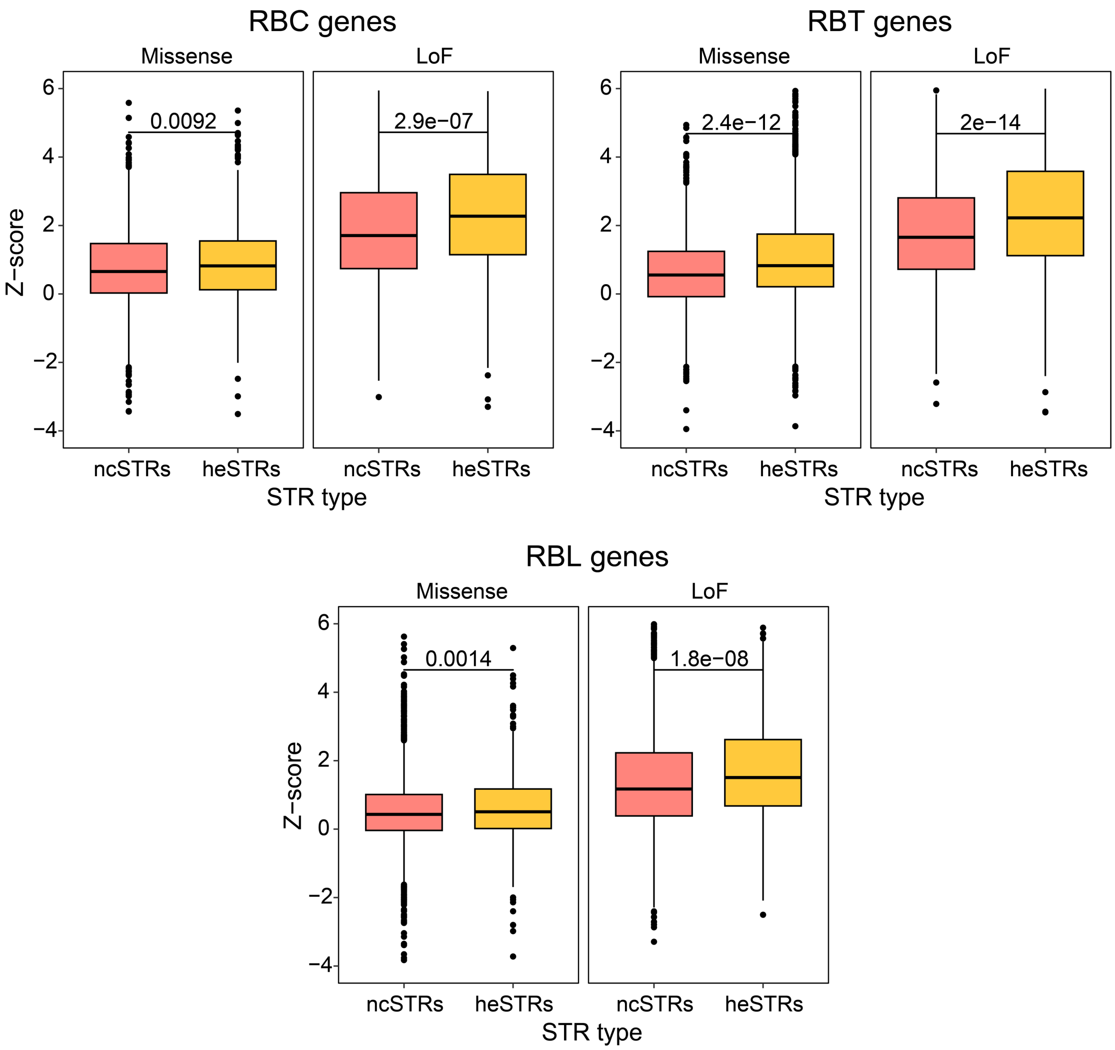

Fig. S16.

Comparison of genomic constraint scores for genes regulated by heSTRs and ncSTRs exclusively through the RBC, RBT, or RBL mechanism. P-values were computed using `wilcox.test` function in R.

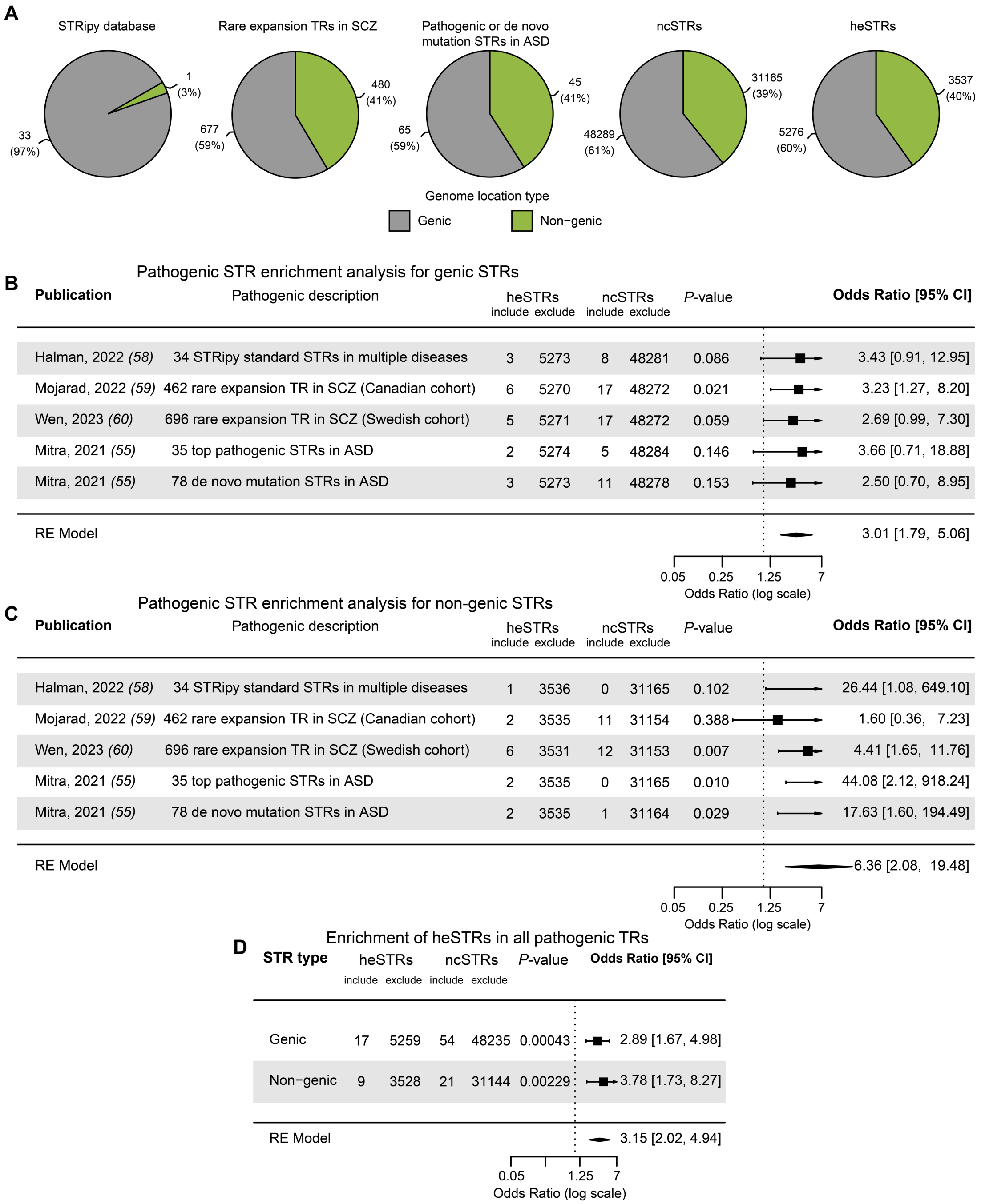

Fig. S17.

Association of genic and non-genic heSTRs with pathogenic STRs. (A), Distribution of genic and non-genic STRs in pathogenic STRs from the STRipy database, two SCZ cohort studies, two ASD datasets, as well as in heSTRs and ncSTRs. (B), Overlap of heSTRs with pathogenic STRs from the four datasets in genic regions. (C), Overlap of heSTRs with pathogenic STRs from the four datasets in non-genic regions. (D), Overlap of heSTRs with combined pathogenic STRs from all four datasets in both genic and non-genic regions. P-values were computed using `fisher.test` function in R.

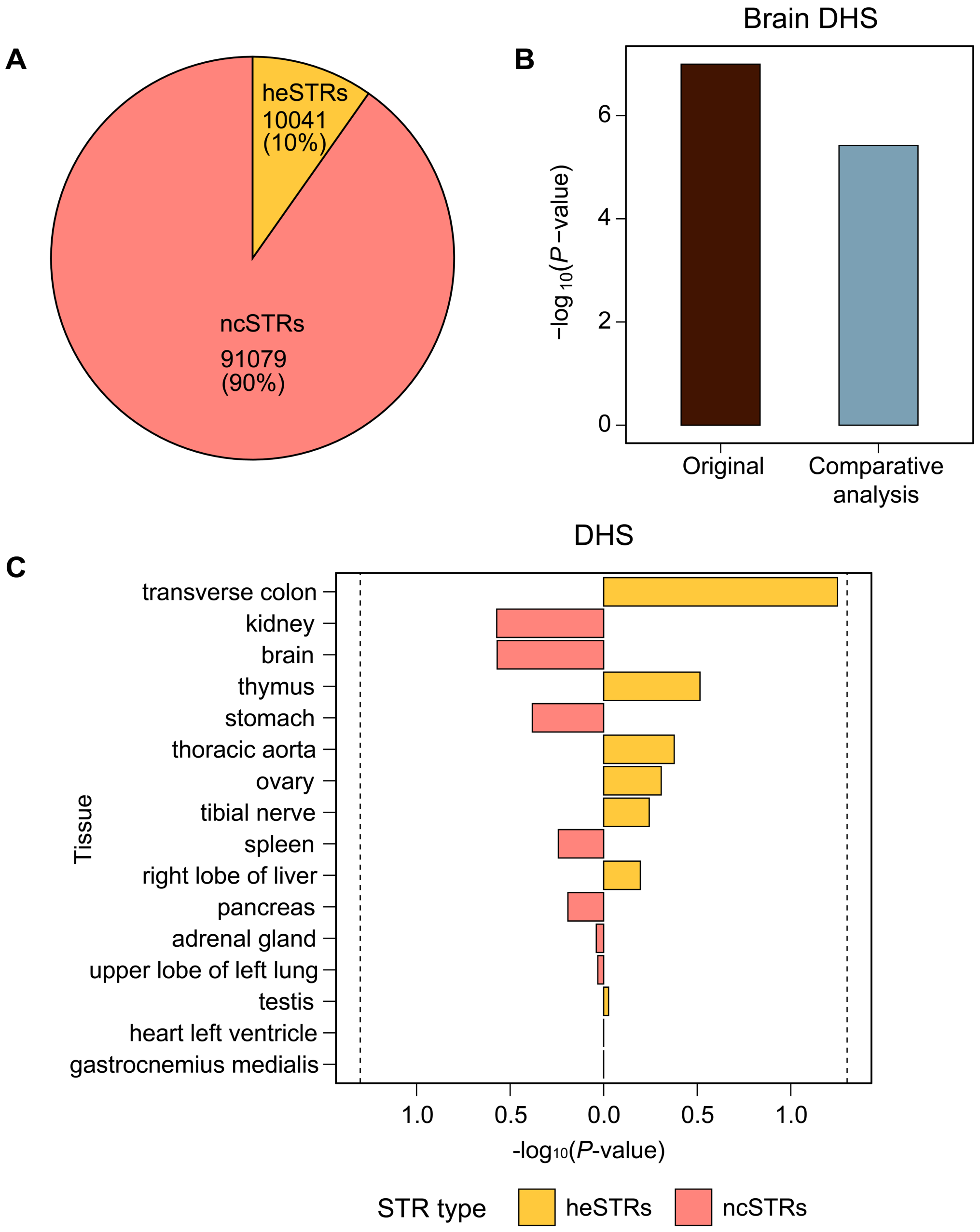

Fig. S18.

Demonstration of the importance of including the three distant NHP species in the evolutionary background when identifying heSTRs. (A), The pie chart displays the number of heSTRs obtained by excluding the three distant NHP species in the evolutionary background. (B), Significance of enrichment in brain DHSs for heSTRs obtained with and without the three distant NHP species in the evolutionary background. (C), Significance of the overlaps between those heSTRs newly identified by excluding the three distant NHP species and DHSs from different tissues. DHSs overlapping with newly identified heSTRs exhibit no tissue specificity.

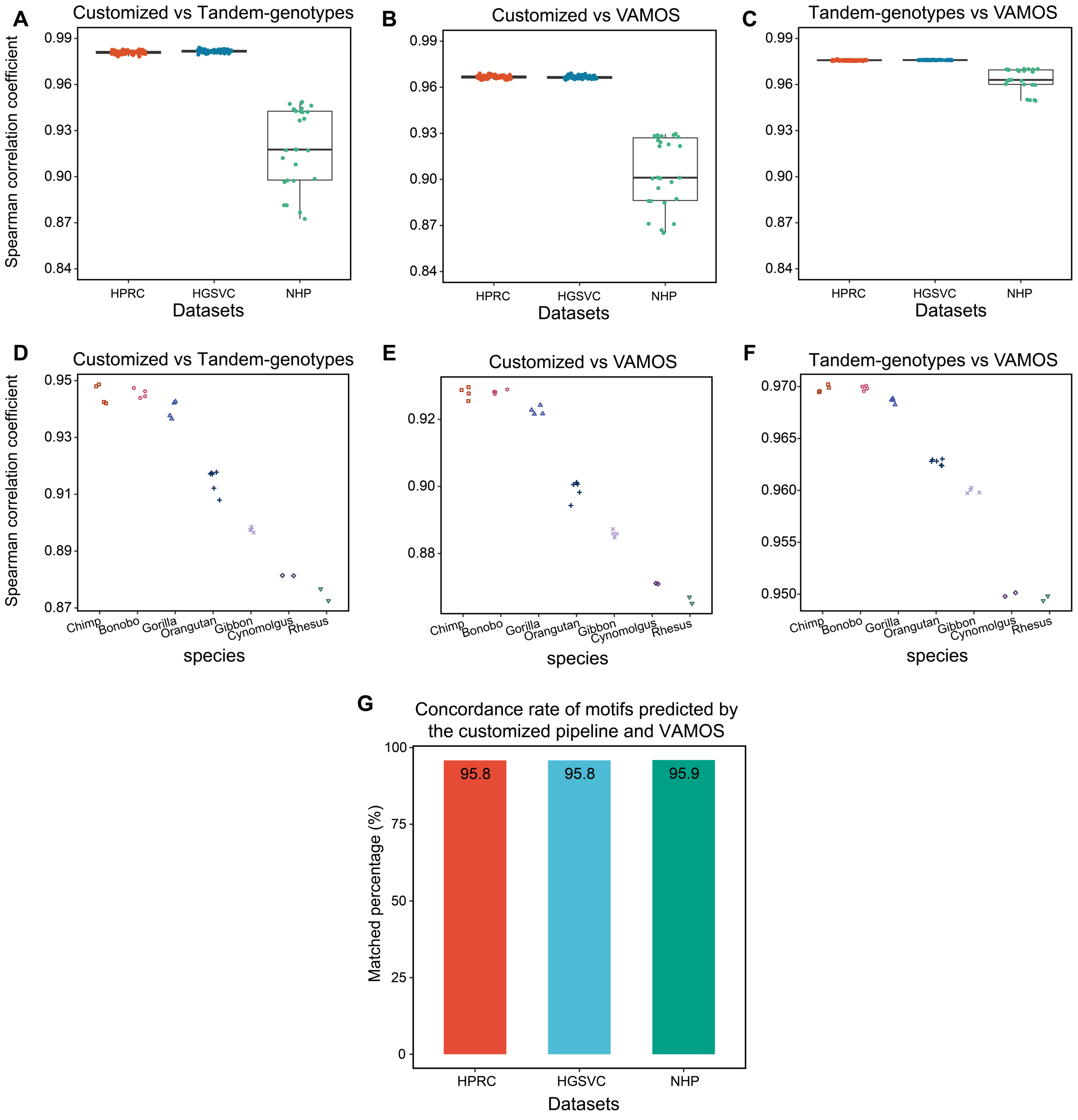

Fig. S19.

Comparison of our customized pipeline with Tandem-genotypes and VAMOS for STR identification. (A-C), Spearman correlation coefficients between STR copy numbers identified by the different methods. (D-F), Spearman correlation coefficients between STR copy numbers identified by the different methods, stratified by evolutionary distances. (G), Concordance rate between motifs predicted by our customized pipeline and VAMOS.

Supplementary Table 1. Information on human and NHP genome samples used in this study.

Supplementary Table 2. Copy number ranges and target genes of 8813 identified heSTRs.

Supplementary Table 3. Resource obtained from ENCODE Project, including ccREs, DHSs, Hi-C data and loop annotations.

Supplementary Table 4. Download links and accessions of the collected cross-species expression datasets.
